# Inhibition of mRNA nuclear export promotes SARS-CoV-2 pathogenesis

**DOI:** 10.1073/pnas.2314166121

**Published:** 2024-05-20

**Authors:** Menghan Mei, Anastasija Cupic, Lisa Miorin, Chengjin Ye, Tolga Cagatay, Ke Zhang, Komal Patel, Natalie Wilson, W. Hayes McDonald, Nicholas A. Crossland, Ming Lo, Magdalena Rutkowska, Sadaf Aslam, Ignacio Mena, Luis Martinez-Sobrido, Yi Ren, Adolfo García-Sastre, Beatriz M. A. Fontoura

**Affiliations:** ^a^Department of Biochemistry, Center for Structural Biology, Vanderbilt University School of Medicine, Nashville, TN 37232; ^b^Department of Microbiology, Icahn School of Medicine at Mount Sinai, New York, NY 10029; ^c^Graduate School of Biomedical Sciences, Icahn School of Medicine at Mount Sinai, New York, NY 10029; ^d^Global Health and Emerging Pathogens Institute, Icahn School of Medicine at Mount Sinai, New York, NY 10029; ^e^Texas Biomedical Research Institute, San Antonio, TX 78227; ^f^Department of Cell Biology, University of Texas Southwestern Medical Center, Dallas, TX 75390; ^g^Shanghai Institute of Immunity and Infection, Chinese Academy of Sciences, Shanghai 200031, China; ^h^Arpirnaut Program, Vanderbilt University School of Medicine, Nashville, TN 37232; ^i^Mass Spectrometry Research Center, Vanderbilt University School of Medicine, Nashville, TN 37232; ^j^Comparative Pathology Laboratory, National Emerging Infectious Diseases Laboratories, Boston University, Boston, MA 02215; ^k^Department of Pathology and Laboratory Medicine, Boston University School of Medicine, Boston, MA 02118; ^l^Department of Medicine, Division of Infectious Diseases, Icahn School of Medicine at Mount Sinai, New York, NY 10029; ^m^Department of Pathology, Molecular and Cell-Based Medicine, Icahn School of Medicine at Mount Sinai, New York, NY 10029; ^n^The Tisch Cancer Institute, Icahn School of Medicine at Mount Sinai, New York, NY 10029

**Keywords:** mRNA export, nuclear transport, SARS-CoV-2, NXF1, Nsp1

## Abstract

The nonstructural protein 1 (Nsp1) of SARS-CoV-2 (Severe Acute Respiratory Syndrome Coronavirus 2) is a virulence factor that targets multiple cellular pathways to inhibit host gene expression and antiviral response. However, the underlying mechanisms of the various Nsp1-mediated functions and their contributions to SARS-CoV-2 virulence remain unclear. Among the targets of Nsp1 is the mRNA (messenger ribonucleic acid) export receptor NXF1-NXT1, which mediates nuclear export of mRNAs from the nucleus to the cytoplasm. Based on Nsp1 crystal structure, we generated mutants on Nsp1 surfaces and identified an acidic N-terminal patch that is critical for interaction with NXF1-NXT1. Photoactivatable Nsp1 probe reveals the RNA Recognition Motif (RRM) domain of NXF1 as an Nsp1 N-terminal binding site. By mutating the Nsp1 N-terminal acidic patch, we identified a separation-of-function mutant of Nsp1 that retains its translation inhibitory function but substantially loses its interaction with NXF1 and reverts Nsp1-mediated mRNA export inhibition. We then generated a recombinant (r)SARS-CoV-2 mutant on the Nsp1 N-terminal acidic patch and found that this surface is key to promote NXF1 binding and inhibition of host mRNA nuclear export, viral replication, and pathogenicity in vivo. Thus, these findings provide a mechanistic understanding of Nsp1-mediated mRNA export inhibition and establish the importance of this pathway in the virulence of SARS-CoV-2.

## Significance

This study shows the impact of inhibiting the mRNA nuclear export machinery on SARS-CoV-2 pathogenesis. By defining the mechanism and interaction surface between the SARS-CoV-2 Nsp1 protein and the mRNA export receptor NXF1, which mediates inhibition of cellular mRNA nuclear export, we were able to generate a mutant SARS-CoV-2 that does not inhibit mRNA nuclear export and is attenuated. Our results establish the importance of Nsp1-mediated mRNA export inhibition for SARS-CoV-2 replication and pathogenesis and reveal a surface on Nsp1 protein that can be potentially targeted for antiviral development.

Inhibition of host gene expression is an important strategy employed by diverse viruses to facilitate their replication, including SARS-CoV-2 which has caused huge impacts on global health and the economy. This strategy allows viruses to not only seize the cellular resources to produce viral proteins but also suppress host immune response by inhibiting the expression of antiviral factors. The nonstructural protein 1 (Nsp1) of SARS-CoV-2 is a key virulence factor that acts at multiple levels to inhibit host gene expression (1–3). Nsp1 is a 20 kDa protein encoded at the very 5′ end of the SARS-CoV-2 RNA genome. It is well characterized that Nsp1 targets the ribosome 40S subunit to plug the mRNA entry channel and thereby inhibit protein translation (2, 4–6). Moreover, Nsp1 has been shown to induce mRNA decay (1) and this effect requires mRNA association with the ribosome (7). We have previously uncovered an unknown Nsp1 function as an inhibitor of host mRNA nuclear export (3). We demonstrated that Nsp1 directly targets the host mRNA export receptor, the NXF1-NXT1 heterodimer, which provides mature mRNAs with access to the nuclear pore complex (NPC) for their nuclear export. As a result, SARS-CoV-2 infection reduces NXF1 docking at the NPC and causes nuclear retention of cellular mRNAs (3). Others have also reported on the mRNA export inhibition by Nsp1 during SARS-CoV-2 infection (1, 8).

mRNA nuclear export is an obligatory step for gene expression in all eukaryotic cells (9, 10). We previously reported that influenza A virus NS1 protein also targets the NXF1-NXT1 complex to inhibit host mRNA nuclear export (11, 12). In particular, NS1 recognizes the NPC binding domain of NXF1 and mimics how NPC interacts with NXF1-NXT1. Consequently, NS1 prevents binding of NXF1-NXT1 to the NPC. Influenza A virus carrying a mutant NS1 incapable of binding to NXF1-NXT1 is attenuated (11). With respect to SARS-CoV-2, Nsp1 acts at a different step during the NXF1-NXT1-mediated process (3). NXF1-NXT1 itself is capable of binding to RNA in vitro, but its loading onto mRNAs in vivo requires the action of the TRanscription-EXport (TREX) complex (13, 14). Nsp1 decreased the association of the TREX complex component Aly/REF to NXF1-NXT1, implying that Nsp1 may prevent the proper configuration of NXF1-NXT1 for the assembly of a productive RNA export complex (3) and/or disrupt both nuclear import and export of NXF1. The molecular mechanism underlying the SARS-CoV-2 Nsp1 mediated mRNA export inhibition, however, remains unknown.

Considering the growing list of SARS-CoV-2 Nsp1’s targets in host cells, including ribosome, NXF1-NXT1, and DNA polymerase α-primase (3, 15, 16), understanding the underlying molecular interactions is crucial to design separation-of-function Nsp1 mutants to dissect the impacts of Nsp1 on different cellular pathways in SARS-CoV-2 infected cells and on viral pathogenesis. An example is the Nsp1-K164A/H165A mutant that inhibits Nsp1 binding to the ribosome. Cryo-EM structures reveal that K164 and H165 reside on the binding interface between Nsp1 and the 40S ribosomal subunit (2, 4–6). This mutant serves as a valuable tool in the studies of Nsp1 translation shutoff because it specifically disrupts the Nsp1-ribosome interaction. To date, there are no available Nsp1 mutants to perturb the Nsp1 and NXF1-NXT1 interaction for further functional studies.

Here, we identify the key regions of Nsp1 that mediate NXF1-NXT1 recognition and are responsible for Nsp1-mediated mRNA export inhibition. We found that both the N-terminal (Nsp1-N) and C-terminal (Nsp1-C) domains of Nsp1 are involved in NXF1-NXT1 interaction. We demonstrate that Nsp1-N employs a patch of acidic amino acids (D33, E36, E37, and E41) for binding to NXF1-NXT1. Using a photoactivatable Nsp1 probe, we show that this Nsp1 acidic patch interfaces with a basic patch on the RNA Recognition Motif (RRM) of NXF1. Mutation on the Nsp1 acidic patch compromised its ability to block mRNA nuclear export but did not affect Nsp1’s inhibitory function in protein translation, indicating this is a separation-of-function mutation. Furthermore, we generated a recombinant (r)SARS-CoV-2 with mutations in the N-terminal acidic patch of Nsp1 and show that this mutant virus is unable to inhibit mRNA export and is attenuated in cells and mice. In fact, the mutant Nsp1 does not properly interact with NXF1 during infection. Together, these findings point to a key function for Nsp1-NXF1 interaction in promoting viral replication and pathogenesis by inhibiting the mRNA export machinery.

## Results

### An Acidic Patch on the Nsp1-N Terminal Domain Is Critical for NXF1-NXT1 Interaction.

**Fig. 1.**
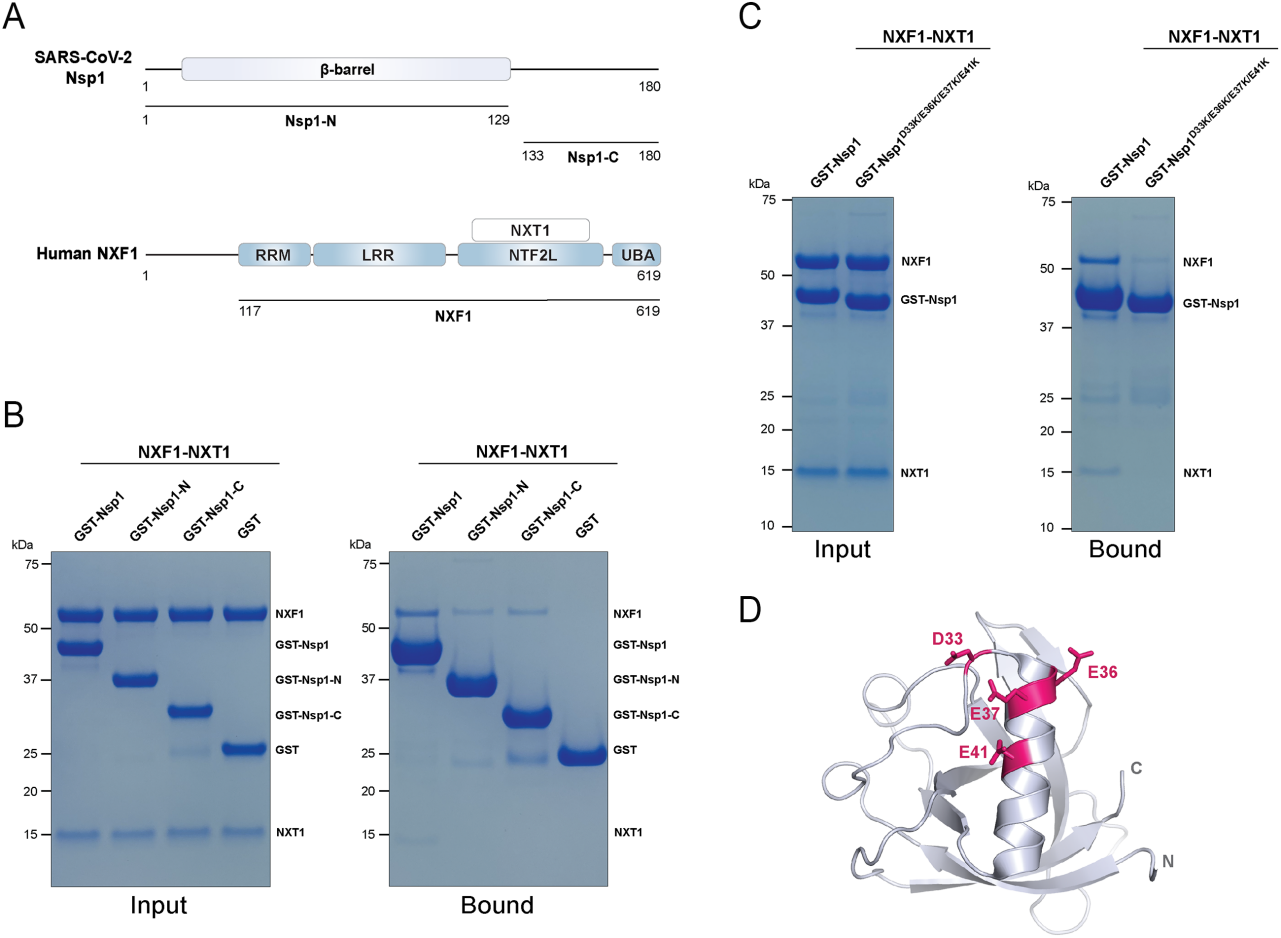
An acidic patch on the Nsp1 N-terminal domain plays a key role in NXF1-NXT1 binding. (*A*) Schematic representation of SARS-CoV-2 Nsp1 and human NXF1-NXT1. (*B*) Both the N-terminal and C-terminal domains of Nsp1 are involved in NXF1-NXT1 binding. In vitro GST pull-down assays were performed with purified GST-tagged Nsp1 variants and NXF1-NXT1. (*C*) Mutations of an acidic patch on Nsp1-N significantly reduced NXF1-NXT1 binding. (*D*) Cartoon representation of Nsp1-N (PDB ID 7K7P) highlighting the acidic patch on Nsp1-N.

Our previous studies established that SARS-CoV-2 Nsp1 inhibits host mRNA export by targeting the mRNA export receptor NXF1-NXT1 complex (3). Here, we sought to determine the mechanisms of the interaction between Nsp1 and NXF1-NXT1. The Nsp1 protein contains an N-terminal globular domain (Nsp1-N) and a C-terminal domain (Nsp1-C). In previous studies, we showed that Nsp1-Full Length (FL, residues 1 to 180) and Nsp1-N (residues 1 to 129) bind to NXF1-NXT1 (3). The contribution of Nsp1-C to NXF1-NXT1 interaction was not examined. We cloned a GST-tagged Nsp1-C (residues 133-180) and used purified recombinant proteins to test interaction with NXF1-NXT1 by GST pull down. We found that each Nsp1-N or Nsp1-C alone is capable of binding to NXF1-NXT1, but both domains are required for optimal binding (Fig. 1 *A* and *B*).

To probe the mechanism by which Nsp1-N recognizes NXF1-NXT1, we systematically mutated Nsp1-N based on its crystal structure and examined the binding between Nsp1 mutants and NXF1-NXT1 (*SI Appendix*, Table S1). The following factors are considered to choose mutation candidates: 1) residues that are solvent exposed, 2) residues with characterized functions (R99A for ribosome binding; R124E/K125E for ribosome binding and promoting RNA degradation (17); L27D/V28D for DNA polymerase α-primase binding) (16), and 3) residues forming charged (i.e. D33K/E36K/E37K/E41K, E55A/E57A/K58E) or hydrophobic patches (L27D/V28D) on the protein surface.

We found that a quadruple point mutant Nsp1^D33K/E36K/E37K/E41K^ substantially reduced NXF1-NXT1 binding compared to wild-type Nsp1 (Fig. 1*C*). Some residual binding remained and may involve Nsp1-C. Nsp1-N folds into a β-barrel capped with an α-helix. D33, E36, E37, and E41 are localized at or near the capping helix forming a highly acidic patch on the Nsp1-N surface (Fig. 1*D*). The critical role of this acidic patch is consistent with the salt-sensitive nature of the Nsp1 interaction with NXF1-NXT1. Of note, other surface charge mutants of Nsp1 (Nsp1^E55A/E57A/K58E^, Nsp1^E91K/L92A/E93K/I95A^) do not reduce NXF1-NXT1 binding (*SI Appendix*, Table S1).

Several residues on the N-terminal domain of Nsp1 have been previously characterized. For instance, R124 and K125 are highly conserved among β-CoVs and play key roles in Nsp1’s RNA cleavage function (18). Nsp1^R99A^ and Nsp1^R124E/K125E^ mutants were recently shown to reduce Nsp1 binding to ribosome (17). As shown in *SI Appendix*, Fig. S1, these mutants do not affect the interaction between Nsp1 and NXF1-NXT1. In addition, the hydrophobic patch mutant Nsp1^L27D/V28D^, which disrupts Nsp1 interaction with DNA polymerase α-primase (16), does not affect Nsp1 and NXF1-NXT1 interaction. Together, our results indicate that the acidic patch is critical for Nsp1 binding to NXF1-NXT1 and is separated from the well-characterized residues involved in other Nsp1-host interactions at Nsp1-N.

### Photoactivatable Nsp1 Probe Identifies NXF1-RRM As an Nsp1 Binding Site.

**Fig. 2.**
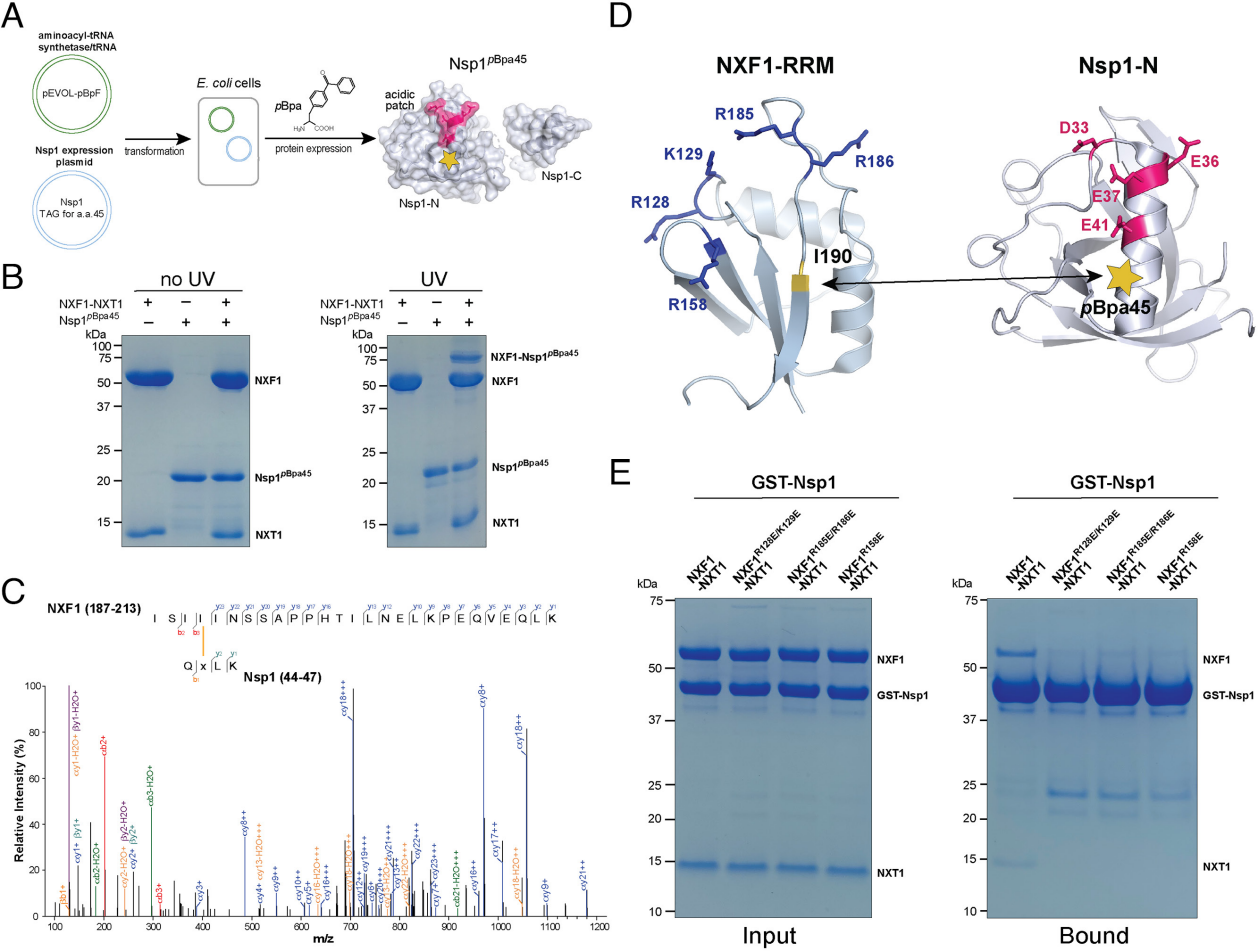
Photoactivatable Nsp1 probe identifies the RRM domain of NXF1 as an Nsp1-N binding site. (*A*) Schematics for incorporation of the photoactivatable nonnatural amino acid *p*Bpa into Nsp1. (*B*) UV treatment of Nsp1*^p^*^BPa45^ in the presence of NXF1-NXT1 generated a new band consistent with the size of crosslinked Nsp1*^p^*^BPa45^-NXF1 complex on SDS-PAGE gel. (*C*) Tandem mass spectrum for a peptide crosslinked to I190 of the NXF1-RRM domain. (*D*) Cartoon representation of NXF1-RRM and Nsp1-N. Positively charged residues (blue) are enriched on the surface of NXF1-RRM forming a basic patch. The yellow star denotes the position of the incorporated *p*Bpa at residue 45. The crosslinking site between Nsp1*^p^*^BPa45^ and NXF1-NXT1 is labeled. (*E*) Mutations of the basic patch residues on NXF1-RRM reduced Nsp1 interaction. In vitro GST pull-down assays were performed with GST-tagged Nsp1 and NXF1-NXT1 variants.

Since Nsp1^D33K/E36K/E37K/E41K^ is a potential candidate for separation-of-function mutant for mRNA export, we focused on further characterizing the interaction between Nsp1-N and NXF1-NXT1. We sought to determine the Nsp1-N binding site on NXF1 using a photo-activatable Nsp1 probe (Fig. 2*A*). We incorporated a photo-crosslinking amino acid *p*-benzoyl-L-phenylalanine (*p*Bpa) to replace His45 on the Nsp1 surface (Nsp1*^p^*^BPa45^) (19). His45 was chosen because it is near the Nsp1-N acidic patch. There is a large chance that this residue is in close proximity to NXF1 in the Nsp1-NXF1-NXT1 complex to allow crosslinking. To generate Nsp1*^p^*^BPa45^, Nsp1 plasmid with His45 replaced by an amber codon was introduced together in *Escherichia coli* with a plasmid carrying an engineered aminoacyl-tRNA synthesis/tRNA pair that incorporates *p*Bpa into proteins in response to the amber codon. We successfully expressed Nsp1*^p^*^BPa45^ in *E. coli* and purified the protein.

For photocrosslinking, Nsp1*^p^*^BPa45^ or NXF1-NXT1 or their mixture was mixed and treated with UV light at 365 nm. A new band corresponding to the size of the crosslinked Nsp1*^p^*^BPa45^-NXF1 complex was observed in the mixture of Nsp1*^p^*^BPa45^ and NXF1-NXT1 on an SDS-PAGE gel (Fig. 2*B*). The SDS-PAGE gel band of the putative Nsp1*^p^*^BPa45^-NXF1 complex from the UV treated mixture was excised and subjected to trypsin digestion and mass spectrometry analysis. A total of 21 interprotein crosslinks were identified with all mapped to the RRM domain of NXF1 (*SI Appendix,* Table S2). Of note, 12 crosslinks are mapped to the ^187^[ISIIINSSAPPHTILNELKPEQVEQLK]^213^ peptide of NXF1-RRM and 7 crosslinks are mapped to the same amino acid I190 within this peptide (Fig. 2*C*).

The RRM domain of NXF1 contains a four-stranded, antiparallel β-sheet and two helices packing against the β-sheet (Fig. 2*D*). The dominant crosslinked residue I190 is located at the β-sheet face. An examination of the surface property of NXF1 RRM shows that the β-sheet face is highly enriched in positively charged residues (R128, K129, R158, R185, and R186). These positively charged residues are highly localized, forming a basic patch on the RRM surface. Considering that Nsp1 employs an acidic patch for binding to NXF1-NXT1, our results strongly suggest that Nsp1 acidic patch interfaces with the basic patch on the NXF1-RRM surface. To examine the contribution of the NXF1-RRM basic patch in Nsp1 binding, we generated three NXF1 mutants, R128E/K129E, R185E/R186E, and R158E. We found that all three basic patch mutants significantly reduced Nsp1 binding (Fig. 2*E*). Some residual binding may involve Nsp1-C. These results demonstrate the critical role of the NXF1-RRM basic patch in binding to Nsp1, which is strongly supported by the crosslinking studies. Together, our crosslinking and mutagenesis studies on NXF1-RRM have two implications. First, residue 45 of Nsp1 is indeed near the interface between Nsp1 and NXF1-NXT1, which reinforces our identification of the Nsp1 acidic patch as a key NXF1-NXT1 binding site. Second, electrostatic interaction involving the basic patch on the RRM domain of NXF1 and the acidic patch on Nsp1 plays a critical role in their interaction.

### The Nsp1 N-Terminal Acidic Patch Is Key for the Inhibitory Function of Nsp1 on Nuclear Export of mRNAs and Not for Inhibition of Translation.

**Fig. 3.**
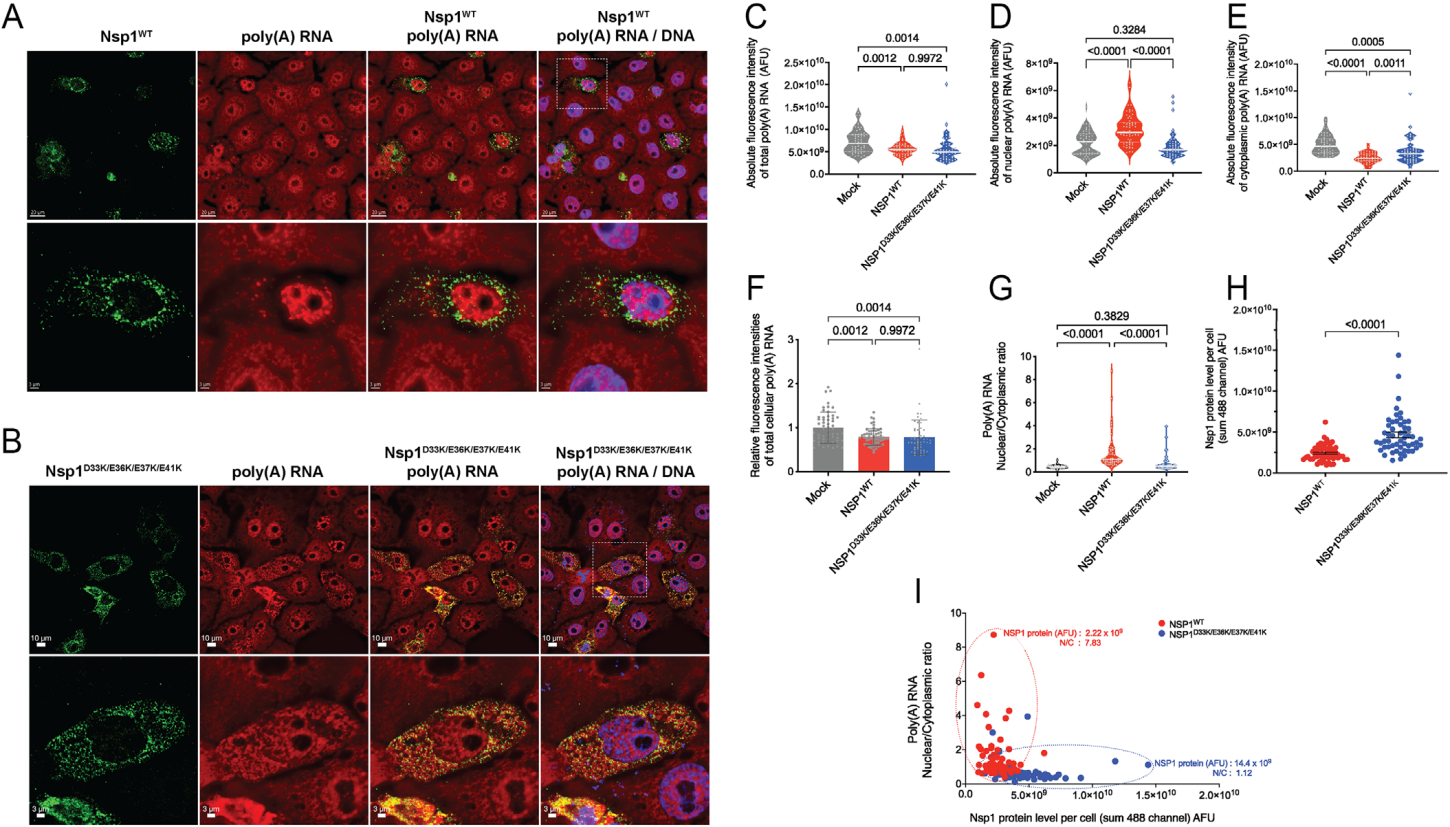
Nsp1 N-terminal mutant D33K/E36K/E37K/E41K can no longer inhibit nuclear export of cellular poly(A) RNA. (*A*) Vero cells were non-transfected or transfected with wild-type^3xFLAG^Nsp1. Nsp1 expression was detected with antibody against the 3xFlag peptide (green) and the intracellular distribution of poly(A) RNA was detected by RNA-FISH using oligo-d(T) probes (red). The same cells were also stained with Hoechst 33258 to visualize nuclei (blue). (Scale bar: 10 µm). Images in the second row correspond to a high magnification of the area surrounded by dotted lines. (Scale bar: 3 µm). (*B*) Vero cells were non-transfected or transfected with mutant ^3xFLAG^Nsp1^D33K/E36K/E37K/E41K^. Cells were processed as in (*A*). (Scale bar: 10 µm.) Second row images correspond to a high magnification of the area surrounded by dotted lines. (Scale bar: 3 µm). Scatter plot representations of absolute fluorescence intensity (arbitrary fluorescence units, AFU) of poly(A) RNA in whole cell (total) (*C*), nucleus (*D*), and cytoplasm (*E*) are shown. Each dot represents a cell. The dashed lines represent quartiles, and the white line represents median value. (*F* and *G*) Relative fluorescence intensity of whole cell (total) cellular poly(A) RNA was quantified in individual cells (*F*) or nuclear to cytoplasmic ratios (N/C) of absolute poly(A) RNA signal (*G*). Data represent three independent experiments. Control, *n* = 54 cells; Nsp1-transfected cells, *n* = 60 cells; Nsp1^D33K/E36K/E37K/E41K^ transfected cells *n* = 53 cells. Image quantification shows the fluorescence intensity of poly(A) RNA from rendered images. Statistical analysis was performed using one‐way ANOVA with a Tukey post test and *P* values are depicted in the figure. (*H*) Scatter plot representations of single cell absolute 488 fluorescence intensity of Nsp1^WT^ or Nsp1^D33K/E36K/E37K/E41K^ expressed in Vero cells with an overall median 488 fluorescence intensity of 2.40 × 10^9^ and 4.66 × 10^9^, respectively. The black lines represent SEM. Statistical analysis was performed using the two-tailed *t* test and *P* value is depicted in the figure. (*I*) Scatter plot showing two dimensions of single infected cells N/C ratios of poly(A) RNA signal versus Nsp1 protein levels (AFU). Clusters of Nsp1^WT^ or Nsp1^D33K/E36K/E37K/E41K^ transfected cells are circled by red or blue dashed lines, respectively. A cell (red) expressing low level of Nsp1^WT^ (2.22 × 10^9^ AFU) and high N/C ratio of poly(A) RNA (N/C = 7.83) is compared to another cell (blue) expressing high levels of Nsp1^D33K/E36K/E37K/E41K^ (14.4 × 10^9^ AFU) and low N/C ratio of poly(A) RNA (N/C = 1.12).

Next, we tested the effect of Nsp1^D33K/E36K/E37K/E41K^ on nuclear export of host mRNA. To this end, cells were transfected with wild-type Nsp1 or with the Nsp1 mutant for 16 h, and the intracellular distribution of poly(A) RNA was determined by fluorescence in situ hybridization (FISH) combined with immunofluorescence microscopy to detect the Nsp1 proteins (Fig. 3 *A–G*). We observed a decrease in whole cell (total) intracellular levels of poly(A) RNA upon expression of the wild-type or mutant Nsp1 proteins (Fig. 3 *C* and *F*). Additionally, cells expressing wild-type Nsp1 showed an increase in the absolute or relative nuclear fluorescence intensity of poly(A) RNA, suggesting nuclear retention of poly(A) RNA (Fig. 3 *D* and *G*). In contrast, absolute or relative nuclear fluorescence intensity of poly(A) RNA did not increase in cells transfected with Nsp1^D33K/E36K/E37K/E41K^, indicating no nuclear export inhibition of mRNA (Fig. 3 *D* and *G*). Expression levels of wild-type and mutant Nsp1 proteins were quantified and showed that although Nsp1 mutant is expressed at higher levels than the wild-type protein (Fig. 3*H*), it is unable to block mRNA export (Fig. 3*I*). These results suggest that mutation of the acidic patch disrupted Nsp1-mediated inhibition of mRNA export.

**Fig. 4.**
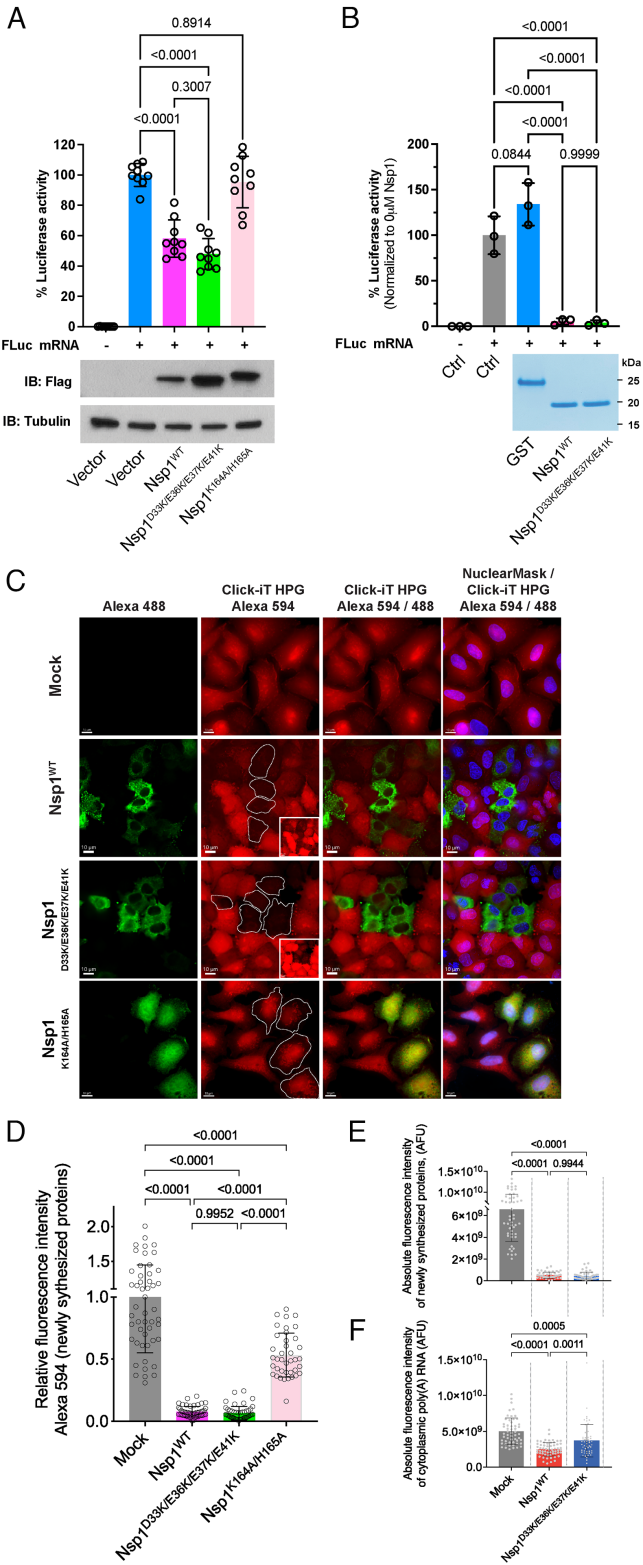
SARS-CoV-2 Nsp1^D33K/E36K/E37K/E41K^ maintains the ability to inhibit translation. (*A*) HEK293T cells were transfected with pCl-neo vector or the indicated pCl-neo SARS-CoV-2 Nsp1 expressing plasmids. Firefly luciferase (Fluc) mRNA was transfected 24 h after DNA transfection. Cells were lysed 10 h after mRNA transfection and luciferase activity was measured. Normalized Nsp1 wildtype or mutant luciferase values were compared to empty vector using a one-way ANOVA with multiple comparisons using Tukey’s correction; *P* values are shown. Cell lysates from the translation assay above were analyzed by western blot to detect Nsp1 (IB: Flag). Tubulin was used as loading control. (*B*) Cell-free in vitro translation assay in rabbit reticulocytes was performed with a capped Fluc reporter mRNA in the presence of GST, Nsp1^WT^, or Nsp1^D33K/E36K/E37K/E41K^ proteins (1 μM). Control reactions were performed in the absence of Nsp1 (Ctrl) with and without capped Fluc mRNA, as well as in the presence of GST (1 μM). To determine statistical significance, one-way ANOVA with multiple comparisons using Dunnett’s correction was performed; p-values are shown. Coomassie-stained SDS-PAGE of GST, Nsp1^WT^, and Nsp1^D33K/E36K/E37K/E41K^ proteins at equal molar amount. (*C*) Nascent protein synthesis was monitored using Click-iT HPG Alexa 594 in A549 cells transfected with plasmids encoding Nsp1^WT^, Nsp1^D33K/E36K/E37K/E41K^, or Nsp1^K164A/H165A^. Cells were pulse-labeled with 0.5 mM HPG for 30 min and chased for 30 min followed by Click-iT™ HPG Alexa Fluor™ 594 protein synthesis assay. Nsp1 expression was detected with antibody against the 3xFlag peptide (green) and Hoechst 33258 was used to visualize nuclei (blue). Transfected cells are marked by dotted lines. *Inset* images in the second column are high-contrast overexposed representation of the corresponding Click-IT HPG Alexa 954 images of Nsp1^WT^ or Nsp1^D33K/E36K/E37K/E41K^ transfected cells to show the low levels of translation activity in these Nsp1 transfected cells. (Scale bar: 10 μm). (*D*) Relative fluorescence intensity of nascent cellular protein synthesis. Single-cell values are shown as open-circle. Bar graphs represent mean values ± SD. (*E*) Absolute fluorescence intensity (arbitrary fluorescence units, AFU) of global proteins synthesis. Each dot shows single-cell measurements. Bar graphs represent mean values ± SD. (*F*) Absolute cytoplasmic poly(A) RNA fluorescence intensity shown in Fig. 3*F*. Each dot represents single-cell quantification. Bar graphs represent mean values ± SD. Data represent three independent experiments. Control, *n* = 50 cells; Nsp1^WT^ transfected cells, *n* = 50 cells; Nsp1^D33K/E36K/E37K/E41K^ transfected cells, *n* = 50 cells; Nsp1^K164A/H165A^ transfected cells *n* = 39 cells.

We then assessed translation in the absence or presence of wild-type Nsp1 or mutant Nsp1 proteins. Cells were cotransfected with capped firefly luciferase reporter mRNA and plasmids encoding wild-type Nsp1, or Nsp1 mutant in the acidic patch, or Nsp1 C-terminal mutant (K164A/H165A). The latter is known to abolish Nsp1-mediated translation inhibition or mRNA degradation (20). While the Nsp1 C-terminal mutant (K164A/H165A) was unable to inhibit translation, as expected, the Nsp1 mutant on the N-terminal acidic patch inhibited translation at similar levels as the wild-type Nsp1 protein (Fig. 4*A*). Notably, in agreement with the release of the mRNA export block, when expressed from a plasmid, the mutant Nsp1 protein is expressed at higher levels than the wild-type protein, indicating that Nsp1 inhibits its own synthesis (Fig. 4*A*). To measure translation in the presence of equal amounts of Nsp1, we then performed a cell-free in vitro translation assay with reticulocyte lysate using purified Nsp1^WT^ or Nsp1^D33K/E36K/E37K/E41K^ proteins. We found that wild-type and mutant Nsp1 proteins inhibit translation of the luciferase reporter mRNA to the same extent in this system (Fig. 4*B*). Additionally, we performed the fluorescent noncanonical amino acid tagging (FUNCAT) assay to assess the effect of wild-type or mutant Nsp1 proteins on translation activity in situ. A549 cells were transfected with plasmids encoding Nsp1^WT^, Nsp1^D33K/E36K/E37K/E41K^, or Nsp1^K164A/H165A^. Cells were pulse-labeled with 0.5 mM HPG for 30 min and chased for 30 min followed by Click-iT™ HPG Alexa Fluor™ 594 protein synthesis assay. Nsp1 expression was detected with antibody against the 3xFlag peptide. We found that both Nsp1^WT^ and Nsp1^D33K/E36K/E37K/E41K^ inhibited nascent cellular protein synthesis to a similar extent whereas the mutant defective in ribosome binding, Nsp1^K164A/H165A^, showed substantial translation activity (Fig. 4 *C* and *D*). We have also compared absolute fluorescence intensity of newly synthesized proteins with the absolute fluorescence intensity of cytoplasmic poly(A) RNA in cells expressing Nsp1^WT^ and Nsp1^D33K/E36K/E37K/E41K^ and found that both robustly inhibited translation even though Nsp1^D33K/E36K/E37K/E41K^ had higher levels of cytoplasmic poly(A) RNA (Fig. 4 *E* and *F*). Taken together, these different approaches corroborate the translation inhibitory activity of Nsp1^D33K/E36K/E37K/E41K^. Thus, this acidic patch Nsp1 mutant is 1) unable to properly interact with the NXF1-NXT1 heterodimer, 2) is also unable to inhibit host mRNA export; however, 3) this Nsp1 mutant maintains its translation inhibitory effect. Together, these results show the identification of a separation-of-function mutation on SARS-CoV-2 Nsp1 protein that can distinguish its effects on translation versus its role in mRNA export.

**Fig. 5.**
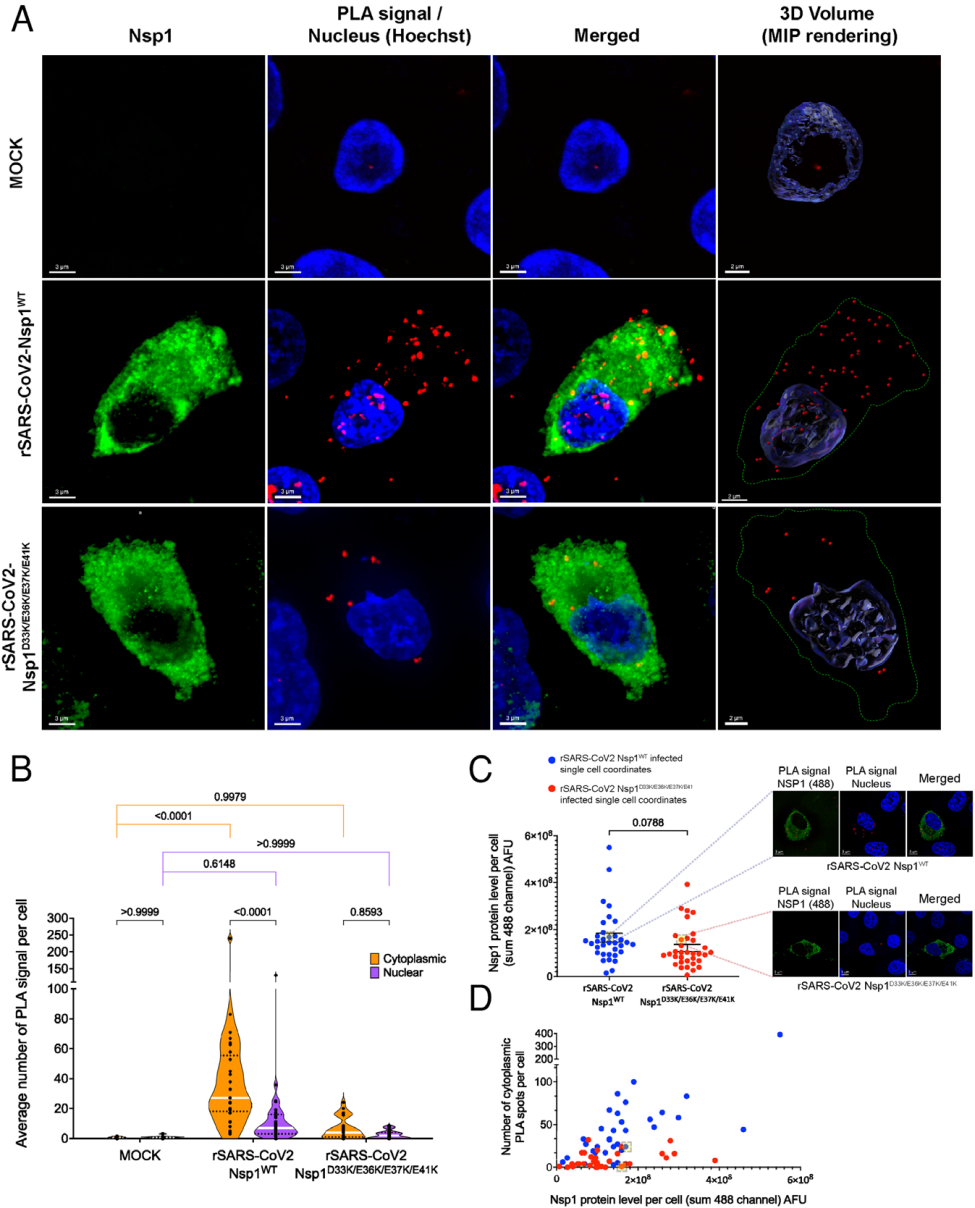
Nsp1-NXF1 interaction is mostly cytoplasmic in rSARS-CoV2^WT^ infected cells and is decreased in rSARS-CoV2^D33K/E36K/E37K/E41K^ infected cells. (*A*) A549-ACE2 cells were mock infected or infected with rSARS-CoV2^WT^ or rSARS-CoV2^D33K/E36K/E37K/E41K^. After 12 h, cells were subjected to PLA to detect the interaction between Nsp1 and NXF1 proteins in situ. The interaction by PLA is detected by fluorescent probes (red dots; λem = 624 nm, TRITC filter). Immunofluorescence staining of Nsp1 is shown in green. Hoechst staining labels the nuclei (blue). (Scale bar: 3 μm). The panels on the *Right* are three-dimensional projections of chromatin merged surface with the PLA signals detecting the NXF1–Nsp1 complexes. Cell boundaries are marked by green-dotted lines. (Scale bar: 2 μm). (*B*) Quantification of PLA signals per cell from 34 cells. The dashed lines represent quartiles and the white line represents median value. Data are representative of three independent experiments. Mean cytoplasmic/nuclear PLA dots per cell are mock: 0.4956/0.8169; rSARS-CoV-2^WT^: 41.29/13.78; rSARS-CoV-2^D33K/E36K/E37K/E41K^: 6.655/2.466. Statistical analysis was performed using one‐way ANOVA with a Tukey post test and *P* values are depicted in the figure. (*C*) Scatter plot representations of single cell absolute 488 fluorescence intensity of Nsp1 protein levels in infected cells. The black lines represent SEM. Single infected cells expressing similar levels of Nsp1 protein (Nsp1^WT^= 1.68 × 10^8^ and Nsp1^D33K/E36K/E37K/E41K^= 1.57 × 10^8^) are shown in the yellow-filled gray brackets with dashed lines projecting on the right to depict their corresponding PLA images (24 PLA dots for rSARS-CoV-2 Nsp1^WT^ infected cell and 1 PLA dot for rSARS-CoV-2 Nsp1^D33K/E36K/E37K/E41K^ infected cell). Statistical analysis was performed using the two-tailed *t* test and *P* value is depicted in the figure. (*D*) Scatter plot shows two-dimensional analysis of individual cells—cytoplasmic PLA spots versus Nsp1 protein level—from rSARS-CoV-2 Nsp1^WT^ or rSARS-CoV-2 Nsp1^D33K/E36K/E37K/E41K^ infected cells. Cells in the yellow-filled gray brackets are the same cells selected in (*C*).

To then assess the importance of the Nsp1 N-terminal acidic patch on its function as inhibitor of host mRNA export during infection, we generated a recombinant (r)SARS-CoV-2 containing the D33K/E36K/E37K/E41K mutation in Nsp1. rSARS-CoV-2 Nsp1^D33K/E36K/E37K/E41K^ was rescued using a bacterial artificial chromosome (BAC)-based reverse genetics system as previously described (21). The presence of the Nsp1^D33K/E36K/E37K/E41K^ mutation was confirmed by genome sequencing of the viral stock (*SI Appendix*, Fig. S2). We then mock-infected or infected cells with rSARS-CoV-2 Nsp1^WT^ or rSARS-CoV-2 Nsp1^D33K/E36K/E37K/E41K^ and subjected the samples to proximity ligation assays (PLAs) to determine Nsp1-NXF1 interaction and their intracellular localization in intact infected cells. In this assay, antibodies that recognize Nsp1 and NXF1 are bound by secondary antibodies linked to oligos that prime a DNA amplification reaction, which is then recognized by fluorescent oligos. The PLA positive signal depicting the interaction between Nsp1 and NXF1 is shown by red dots (Fig. 5*A*). In mock infected cells, only a small number of background dots were detected, demonstrating specificity of the reaction in the infected cells (Fig. 5 *A* and *B*). In cells infected with the wild-type virus, quantification of interactions and their intracellular localization shows that the Nsp1-NXF1 interaction occurs mostly in the cytoplasm but a small pool is also found in the nucleus (Fig. 5 *A* and *B*). In contrast, the interaction between Nsp1^D33K/E36K/E37K/E41K^ and NXF1 was overall reduced in cells infected with this mutant virus, regardless of Nsp1 mutant protein levels (Fig. 5 *C* and *D*). This decrease in binding corroborates the in vitro binding assays shown in Fig. 1. Taken together, these findings indicate that Nsp1 may alter the trafficking of NXF1 into and/or out of the nucleus, which is consistent with Nsp1-mediated inhibition of nuclear export of host mRNAs, as we and others have previously shown (1, 3).

**Fig. 6.**
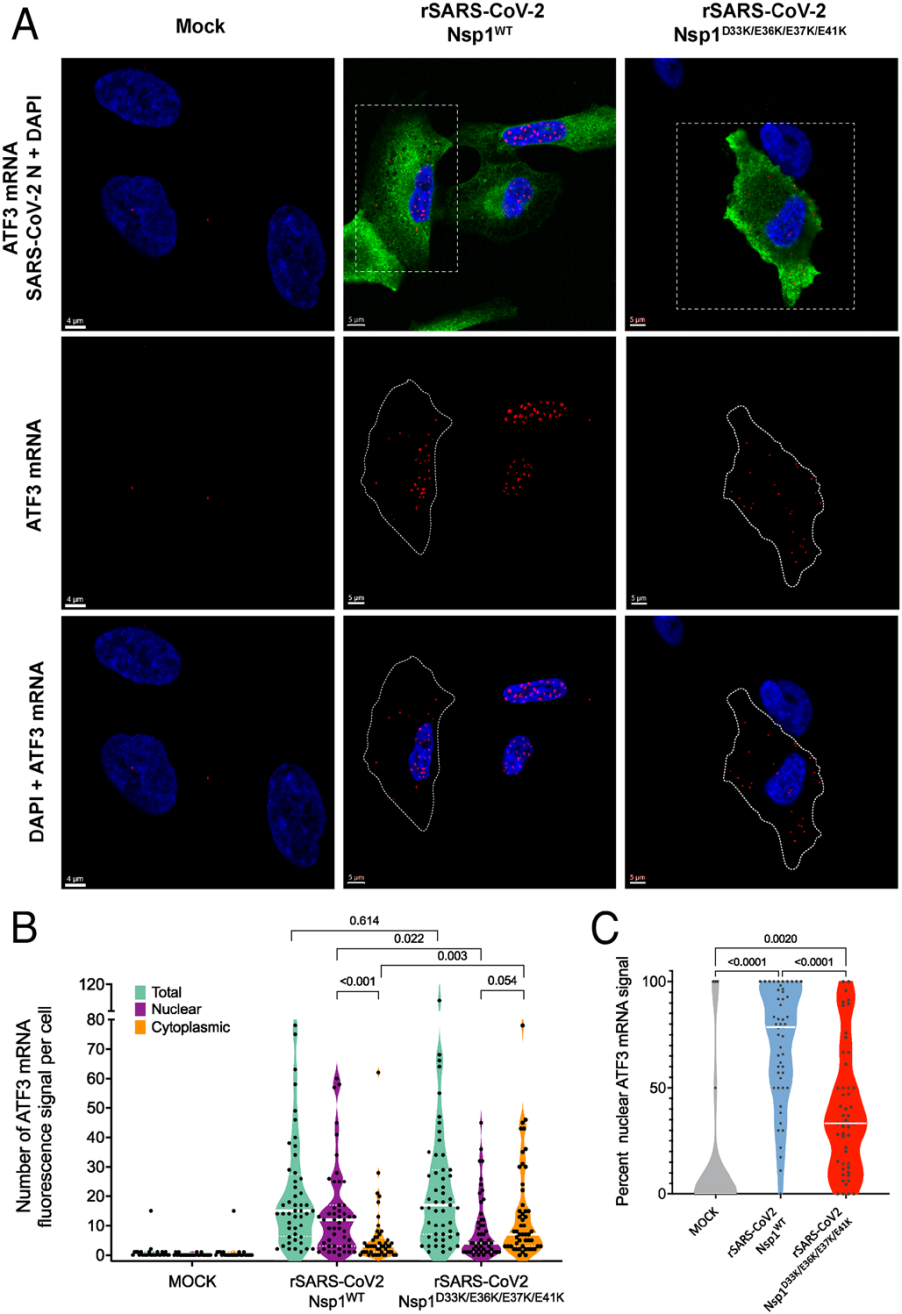
SARS-CoV-2 Nsp1^D33K/E36K/E37K/E41K^ is unable to inhibit cellular mRNA export. A549-ACE2 cells were infected with rSARS-CoV-2 Nsp1^WT^ or rSARS-CoV-2 Nsp1^D33K/E36K/E37K/E41K^ at MOI 0.25 for 24 h. Cells were subjected to the ViewRNA Cell Plus Assay to detect specific cellular mRNAs, as depicted in the figure, and immunofluorescence microscopy to detect viral N protein. (*A*) ATF3 mRNA is detected in red, viral N protein is shown in green, and nuclei are stained in blue with DAPI. The dotted square shows a selected cell that is highlighted in the *Middle* and *Bottom* panels. Images and quantification of other mRNAs are shown in *SI Appendix*, Fig. S3. (*B*) Scatter plot representations of single cell ATF3 mRNA fluorescence signal in whole cells (total), or in the nucleus, or in the cytoplasm of mock, rSARS-CoV-2 Nsp1^WT^, or rSARS-CoV-2 Nsp1^D33K/E36K/E37K/E41K^-infected cells. Each dot represents a cell. The dashed lines represent quartiles and the white line represents median value. (*C*) The calculated percent nuclear ATF3 mRNA signal is presented as a scattered plot for each individual cell (mock n= 25 cells; wild-type virus n= 51 cells; mutant virus n= 51 cells). Each dot represents a cell and the median value is depicted in the white line. Statistical analysis was performed using one-way ANOVA with a Tukey post test and *P* values are depicted in the figure.

Since Nsp1^D33K/E36K/E37K/E41K^ binding to NXF1 is reduced compared to Nsp1 wild-type, we then tested whether these mutations would diminish the inhibitory function of Nsp1 on nuclear export of cellular mRNAs. We performed RNA FISH to detect the intracellular localization of specific cellular mRNAs (ATF3, CXCL3, NFKB1, and NUAK2) previously shown to be retained in the nucleus upon SARS-CoV-2 infection or Nsp1 transfection (1). These mRNAs are present at almost undetectable levels in uninfected cells and once they are transcriptionally induced in the nucleus upon infection, they face the mRNA nuclear export inhibition by Nsp1. While we found that the mRNAs encoding ATF3, CXCL3, and NFKB1 were up-regulated upon rSARS-CoV-2 Nsp1^WT^ infection but were retained inside the nucleus, cells infected with rSARS-CoV-2 Nsp1^D33K/E36K/E37K/E41K^ showed nuclear export of these messages, indicating reversal of mRNA export inhibition by these mutations (Fig. 6 *A–C* and *SI Appendix*, Fig. S3). In the case of NUAK2 mRNA, there is some degree of mRNA retention in the nucleus upon infection with the wild-type virus, which is not significantly different from the infection with the Nsp1 mutant virus, as shown by the intracellular distribution of mRNAs at the single-cell level (*SI Appendix*, Fig. S3 *D* and *K*–*M*). These results suggest differential mRNA export regulation, which is observed in various cellular conditions as diverse mRNAs interact with specific RNA binding proteins and/or mRNA export complexes (22). Taken together, these findings underscore the importance of the N-terminal acidic patch of Nsp1 and its interaction with NXF1 on inhibition of mRNA nuclear export by SARS-CoV-2.

### SARS-CoV-2 Nsp1^D33K/E36K/E37K/E41K^ Is Attenuated at the Cellular and Animal Levels.

**Fig. 7.**
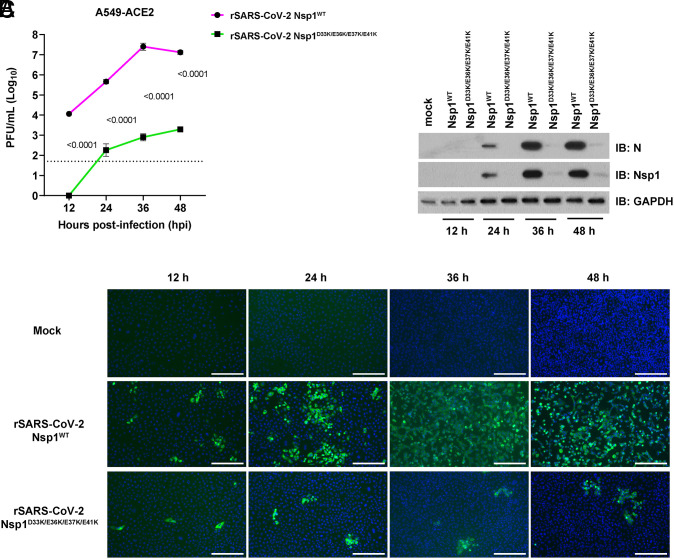
rSARS-CoV-2 Nsp1^D33K/E36K/E37K/E41K^ is attenuated in A549-ACE2 cells. (*A*) Growth curves of rSARS-CoV-2 Nsp1^WT^ and rSARS-CoV-2 Nsp1^D33K/E36K/E37K/E41K^ in A549-ACE2 cells infected at an MOI of 0.1 for 12, 24, 36, and 48 hpi. Titers were quantified by plaque assay (*n* = 3). The dashed line indicates the limit of detection for plaque assay (50 PFU/mL). Values were log transformed and compared using multiple unpaired *t* tests; *P* values are shown. (*B*) A549-ACE2 cells were mock-infected or infected at an MOI of 0.1 and samples were collected at 12, 24, 36, and 48 hpi. Cell lysates were analyzed by Western blot to assess SARS-CoV-2 N and Nsp1 expression. GAPDH was used as loading control. (*C*) A549-ACE2 cells were mock-infected or infected with the indicated viruses at an MOI of 0.1 for 12, 24, 36, and 48 h. Cells were subjected to immunofluorescence microscopy to detect SARS-CoV-2 N protein (green). Nuclei were stained with DAPI (blue). (Scale bar: 300 μm).

We next compared viral replication between the mutant SARS-CoV-2 Nsp1^D33K/E36K/E37K/E41K^ and SARS-CoV-2 Nsp1^WT^. A549-ACE2 cells were infected with wild-type or mutant viruses for 12 h, 24 h, 36 h, and 48 h, and viral replication was determined by plaque assay. Replication of the mutant virus is severely compromised, as shown by its lower viral titers compared to wild-type rSARS-CoV-2 (Fig. 7*A*). Cell lysates were then subjected to Western blot analysis to detect the viral nucleocapsid (N) protein and Nsp1. The mutant virus is clearly attenuated, showing low levels of N and Nsp1 proteins in infected cells (Fig. 7*B*). This is further corroborated by following infection of both wild-type and mutant viruses by immunofluorescence microscopy in which infected cells were stained with antibodies against the N protein (Fig. 7*C*). Again, cells infected with the mutant virus show substantially less N staining than cells infected with wild-type SARS-CoV-2.

**Fig. 8.**
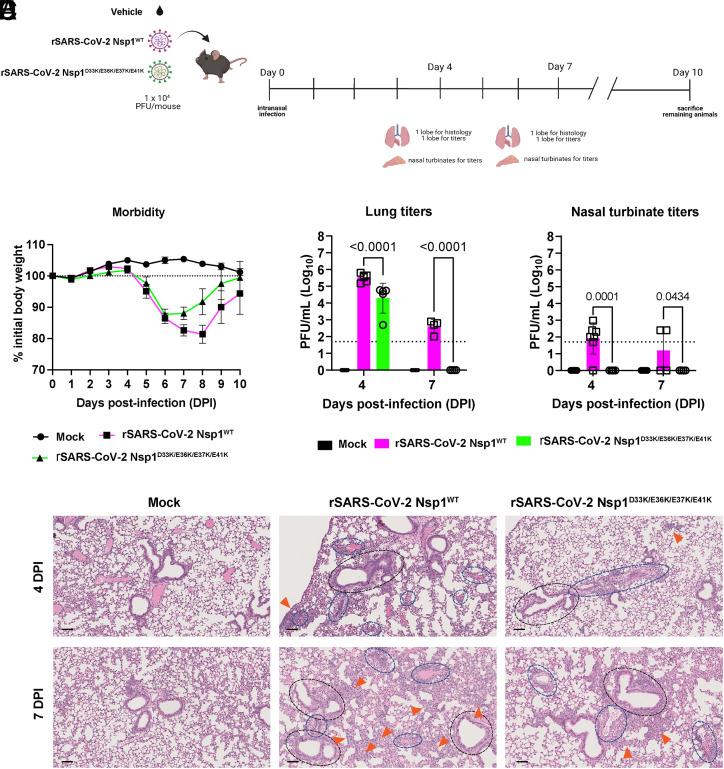
rSARS-CoV-2 Nsp1^D33K/E36K/E37K/E41K^ infected K18-hACE2 mice show less severe lung histopathology. (*A*) Schematic of in vivo experimental design where K18-hACE2 mice were mock-infected (n = 7 at 4 dpi and n = 4 at 7 dpi) or infected with 1 × 10^4^ PFU of rSARS-CoV-2 Nsp1^WT^ (n = 7 at 4 dpi and n = 4 at 7 dpi) or rSARS-CoV-2 Nsp1^D33K/E36K/E37K/E41K^ (n = 5 at 4 dpi and n = 4 at 7 dpi). (*B*) Weight loss curve depicting body weights for mock-infected or infected with rSARS-CoV-2 Nsp1^WT^ or rSARS-CoV-2 Nsp1^D33K/E36K/E37K/E41K^ for the duration of the experiment as a percentage of initial bodyweight. Weight loss data are shown as mean ± SEM. (*C*) Lung viral titers for mock-infected and infected animals at the indicated dpi. (*D*) Nasal turbinate viral titers for mock-infected and infected animals at the indicated dpi. Dashed lines in (*C*) and (*D*) indicate the limit of detection for plaque assay (50 PFU/mL). Values were log transformed and compared using two-way ANOVA with Tukey’s correction; *P* values are shown. (*E*) Representative images of lung H&E staining for all three groups of animals at the indicated dpi. All images were acquired at 200× total magnification. Mock inoculated lungs were within normal limits, while both infected lung samples displayed variable perivascular (blue hashed ovals), peribronchiolar (black hashed ovals), and interstitial mononuclear infiltrates (orange arrowheads). Endothelial hypertrophy was also observed in both infected cohorts. (Scale bars in histology slides = 100 μm).

To then evaluate the contribution of Nsp1-mediated mRNA export block to the pathogenesis of SARS-CoV-2, 8-wk-old female transgenic mice expressing human ACE2 under the human keratin 18 promoter (K18-hACE2) were intranasally mock-infected or infected with 1 × 10^4^ PFU of either parental rSARS-CoV-2 Nsp1^WT^ or rSARS-CoV-2 Nsp1^D33K/E36K/E37K/E41K^. Animals from each group were killed at 4 and 7 days postinfection (dpi) to determine lungs and nasal turbinate titers. In addition, body weight was recorded for 10 dpi to monitor morbidity. As shown in Fig. 8, we observed that animals infected with rSARS-CoV-2 NSP1^D33K/E36K/E37K/E41K^ exhibited reduced body weight loss compared to those infected with the wild-type virus (Fig. 8 *A* and *B*). Additionally, we observed significantly lower viral load in the lungs and nasal turbinate of rSARS-CoV-2 NSP1^D33K/E36K/E37K/E41K^ -infected animals in comparison to those infected with wild-type virus at both 4 and 7 dpi (Fig. 8 *C* and *D*). Notably, all rSARS-CoV-2 NSP1^D33K/E36K/E37K/E41K^ -infected animals had undetectable levels of infectious virus in the nasal turbinate at both 4 and 7 dpi (Fig. 8*D*). Immunohistochemistry (IHC) also showed reduced SARS-CoV-2 N antigen load in the lungs of K18-hACE2 mice infected with rSARS-CoV-2 Nsp1^D33K/E36K/E37K/E41K^ compared to rSARS-CoV-2^WT^ (*SI Appendix*, Fig. S4). All mock-infected mice examined at 4 and 7 dpi were histologically within normal limits with no apparent immune cell infiltrates. K18-hACE2 mice infected with both wild-type and mutant viruses displayed similar lesions but exhibited variable severity ranging from minimal to regionally moderate disease, with the latter being more common in the mice infected with the wild-type virus. Histologic findings consisted of bronchointerstitial and perivascular mononuclear infiltrates and endothelial cell hypertrophy (Fig. 8*E*). Inflammatory cell infiltrates in each anatomical compartment (peribronchiolar, perivascular, and interstitium) as well as overall percentage of pulmonary parenchyma affected were scored using an ordinal scoring system. The most consistent differences occurred in the interstitial and perivascular compartments, which show less inflammation and infiltrate in mice infected with the mutant virus than in mice infected with the wild-type virus, while the peribronchiolar mononuclear infiltrates were routinely minimal to mild in K18-hACE2 mice infected with both viruses (*SI Appendix*, Fig. S5 and Table S3). In sum, these findings underscore the importance of the interaction between Nsp1 and NXF1 in the inhibition of host gene expression via mRNA export inhibition and show its impact in promoting viral replication and pathogenesis.

## Discussion

Nsp1 is a major virulence factor of SARS-CoV-1 (20, 23, 24) and SARS-CoV-2 (1, 3, 25, 26), functioning as inhibitor of host gene expression through multiple mechanisms. Among the key pathways targeted by SARS-CoV-2 Nsp1 is mRNA nuclear export, as we and others recently discovered (1, 3, 8). Despite intensive studies, the underlying mechanisms of the various Nsp1-mediated functions and their contributions to SARS-CoV-2 virulence remain unclear. In this study, we show that Nsp1 uses a distinct mechanism to interact with the cellular mRNA export receptor NXF1. We identified a separation-of-function mutant of Nsp1, which we employed to investigate the role of the Nsp1-NXF1 interaction in SARS-CoV-2 virulence using SARS-CoV-2 infected cells and an in vivo model. Our results provide a mechanistic understanding of the Nsp1-mediated mRNA export inhibition and reveal the critical importance of this mechanism in the virulence of SARS-CoV-2.

Through structure-guided mutagenesis, we identified an acidic patch (D33, E36, E37, and E41) on the surface of the N-terminal domain of Nsp1 that mediates interaction with NXF1. We employed site-specific photo-crosslinking using *p*Bpa-incorporated Nsp1 at a site near the acidic patch to reveal that Nsp1 binds to the RRM domain of NXF1. Among the various Nsp1-mediated mechanisms, it is established from early work on SARS-CoV-1 and recent studies on SARS-CoV-2 that Nsp1 binds to the ribosome (2, 4–6, 15). The Nsp1-ribosome interaction is responsible for the inhibition of host translation (2, 4–6), and Nsp1-mediated mRNA degradation also involves mRNA interaction with the ribosome (7). However, some mRNAs that encode antiviral factors, such as interferon mRNAs, can evade degradation during SARS-CoV-2 infection (27). Nsp1 primarily targets the ribosome with its C-terminal region, which blocks the mRNA entry site on the 40S ribosomal subunit (2, 4–6). More recent studies show that Nsp1-N also contributes to ribosome binding, involving R99, R124, and K125 (17). We show that mutations of these residues do not affect the Nsp1-NXF1 interaction. Recent studies have shown that Nsp1-N terminal domain dynamically interacts with the 40S ribosomal subunit (28). In addition to the extensively characterized high-affinity Nsp1-C terminal domain interaction with the 40S subunit, the Nsp1-N terminal domain from three different β-CoVs (SARS-CoV-2, MERS-CoV, and Bat-Hp-CoV) also contributes to inhibition of translation. Cryo-EM studies using Bat-Hp-CoV Nsp1 as a model system captured the Nsp1-N terminal domain in the context of the 40S–Nsp1 complex. Indeed, the acidic patch mediating NXF1 interaction is not in contact with the 40S subunit in the structure (28). In addition, mutations of Nsp1-N motifs (_98_GRSG_101_ and _124_RK_125_) mediating 40S interaction do not interfere with NXF1 binding, as shown in our GST pull-down assays (*SI Appendix*, Fig. S1, Nsp1 R99A and R124E/K125E). This recent work supports our conclusion that Nsp1-N terminal domain interacts with NXF1 and ribosome via distinct modes and that the acidic patch is a site that can distinguish Nsp1’s effects on mRNA export versus translation. In fact, we demonstrate that the acidic patch mutant of Nsp1 (D33K/E36K/E37K/E41K) is important for the Nsp1-NXF1 interaction but inhibits mRNA translation at similar levels as the wild-type Nsp1, therefore it does not alter Nsp1’s impact on translation. In sum, we observed that the intact N- and C-terminal domains of Nsp1 are both critical for proper interaction of Nsp1 with NXF1-NXT1 and we were able to identify a four amino acid patch in the N terminus of Nsp1 which once mutated disrupts Nsp1 interaction with NXF1-NXT1 without interfering with Nsp1 effect on translation.

Our previous findings showed that NXF1 interactions with mRNA export factors, such as Aly/REF, and nucleoporins decreased in the presence of Nsp1, suggesting that docking and translocation of NXF1-bound mRNAs through the NPC were compromised (3). However, most of Nsp1 is cytoplasmic, and only a small pool is found in the nucleus (3), so determining the localization of the Nsp1-NXF1 complex is key to further understand Nsp1 activity. Here, we demonstrated that NXF1-Nsp1 interaction occurs mostly in the cytoplasm of infected cells, but a small pool of NXF1 bound to Nsp1 is also detected in the nucleus. Mutation of the Nsp1 in the N-terminal acidic patch diminished the interaction in both compartments, further corroborating the in vitro binding assays. These results indicate that Nsp1 may impair the dynamics of NXF1 recycling between the nucleus and the cytoplasm and/or prevent proper import of NXF1 into the nucleus after translation. It is possible that Nsp1 may alter the interaction of NXF1 with karyopherins/importins in the cytoplasm leading to inhibition of nuclear import of NXF1, and in the nucleus, Nsp1 may affect NXF1-binding to mRNA export factors, such as Aly/REF as we have previously shown (3). These effects would certainly lead to impaired nuclear export of mRNAs.

The Nsp1 N-terminal acidic patch mutant shows clear reversal of mRNA export inhibition compared to the wild-type Nsp1 protein without affecting translation inhibition. This separate-of-function mutant also allowed us to assess the impact of Nsp1-mediated mRNA export inhibition on viral replication at the cellular and animal levels. Indeed, Nsp1 inhibition of mRNA export is important for proper SARS-CoV-2 protein expression and replication. These findings are in agreement with our previous report in which increased levels of the mRNA export receptor NXF1 reverted the mRNA export inhibition mediated by Nsp1 and decreased SARS-CoV-2 infection (3). The rescue of mRNA export includes mRNAs that encode proteins such as NFKB1 and ATF3, which are transcription factors that induce expression of genes involved in immunity and other cellular processes (29–31). While both NFKB1 and ATF3 mRNAs are up-regulated during SARS-CoV-2 infection, they are retained in the nucleus. Upon infection with the mutant virus, these mRNAs are efficiently exported to the cytoplasm and at least a certain amount is translated to induce an antiviral response and the observed decrease in viral replication. Under this condition, the translation machinery is inhibited by Nsp1 but not fully blocked as cells are alive. These results further point to the importance of Nsp1-NXF1 interaction in the inhibition of nuclear export of mRNAs with key antiviral activities.

Next, we assessed the impact of Nsp1-NXF1 interaction in the pathogenesis of SARS-CoV-2 infection. Our results demonstrated that targeting the mRNA export machinery is important for efficient viral replication in the respiratory tract. In fact, compared to mice infected with rSARS-CoV-2 Nsp1^WT^, mice infected with rSARS-CoV-2 Nsp1^D33K/E36K/E37K/E41K^ displayed significantly lower viral titers in the lung and undetectable viral titers in the nasal turbinate, suggesting decreased viral replication and faster clearance of infection in the respiratory tract. Histologically, this was reflected by less severe interstitial and perivascular mononuclear infiltration in the lungs of rSARS-CoV-2 NSP1^D33K/E36K/E37K/E41K^ -infected animals. Additionally, a SARS-CoV-2 with a deletion in the Nsp1 C-terminal domain (amino acids 155 to 165), which disrupts translation inhibition, has also been shown to be attenuated (8). Thus, the combinatory effects of Nsp1 on mRNA export and translation yield a robust inhibitory function on host gene expression that down-regulates IFN response and promote viral replication and pathogenesis.

## Materials and Methods

### Cell Culture, Plasmids, and Antibodies.

Vero cells from the American Type Culture Collection were cultured in DMEM (Sigma-Aldrich) supplemented with 10% (v/v) fetal bovine serum (FBS; Sigma-Aldrich) and 1% antibiotic–antimycotic (Life Technologies, Thermo Fisher Scientific) at 37 °C in 5% CO2. Cells were plated at 5 × 10^5^ density per well (containing a sterile No. 1.5 glass coverslip) of a 24-well culture plate (Phenix Research Products). Transfections were performed according to the manufacturer’s instructions (Lipofectamine 3000, Life Technologies, Thermo Fisher Scientific). For all transient transfections, cells were subjected to RNA FISH-Immunofluorescence 16 h post‐transfection. Vero E6 cells expressing ACE2 and TMPRSS2 (Vero AT) were obtained from BEI Resources (NR-54970) and maintained in Dulbecco’s modified Eagle medium (DMEM) supplemented with 10% (v/v) FBS (VWR), 1% penicillin-streptomycin (Corning), and 10 µg/mL puromycin (ThermoFisher Scientific). HEK293T and A549-ACE2 were maintained in Dulbecco’s modified Eagle’s medium (Corning) supplemented with 10% (v/v) FBS (Peak Serum) and penicillin/streptomycin (Corning) at 37 °C and 5% CO_2_. All cell lines were regularly screened for *Mycoplasma* contamination using the Universal *Mycoplasma* Detection Kit (ATCC, 30-1012K) and tested negative. Plasmids and antibodies are described in *SI Appendix*.

### Protein Purification.

GST-NXF1^117–619^-NXT1 wild-type and mutants were coexpressed in *E. coli* Rosetta cells (EMD Millipore, 70954). Protein expression and purification are described in *SI Appendix*.

### GST-Pulldown Assay.

Ten micromolar GST or GST-Nsp1 or GST-Nsp1 variants were mixed with 6 µM NXF1^117–619^-NXT1 wild type or mutant and incubated with Glutathione Sepharose resins in a binding buffer containing 20 mM HEPES pH 7.0, 100 mM NaCl, and 0.5 mM TCEP at room temperature for 40 min. Resins were washed with 600 µL binding buffer for three times. Proteins remaining on the resin were extracted with an elution buffer containing 20 mM HEPES pH 7.0, 300 mM NaCl, 0.5 mM TCEP, and 20 mM reduced glutathione and resolved on a 4 to 12% SDS-PAGE gel. All experiments were repeated three times independently.

### Photo-Crosslinking of NXF1-NXT1 with Nsp1*^p^*^Bpa45^ and LC–MS/MS Analysis.

Ten micromolar NXF1-NXT1 was incubated with 10 µM Nsp1*^p^*^Bpa45^ in a buffer containing 20 mM HEPES pH 7.0, 100 mM NaCl, and 0.5 mM TCEP on ice for 30 min. Protein mixture was irradiated at 365 nm (Spectrolinker; 120V, 60 HZ, 3A) on ice in a 96-well plate (Thermo Fisher Scientific) for 25 min. Samples were resolved on a 15% SDS-PAGE gel.

SDS-PAGE gel bands corresponding to the crosslinked Nsp1*^p^*^Bpa45^-NXF1 complex along with each of the individual uncrosslinked proteins were excised and subjected to in-gel digestion using trypsin. The resulting peptides were analyzed by LC–MS/MS utilizing a QExactive-plus mass spectrometer (ThermoFisher) directly coupled to a nano-flow HPLC (U3000, ThermoFisher) using a self-packed 20 cm × 100 micron C18 column. They were resolved using a 90 min aqueous to organic gradient and MS/MS spectra were collected in a data-dependent manner. Resulting MS/MS spectra were analyzed using the pLink software suite (32) using default parameters with the exception that minimum peptide length was set to 4 to accommodate the shorter *p*BPA-containing peptide. Using a 5% false discovery rate cutoff, a total of 21 interprotein crosslinks were identified. Of these 12 mapped crosslinks to the ISIIINSSAPPHTILNELKPEQVEQLK peptide. Summary of the Nsp1*^p^*^Bpa45^-NXF1 crosslinks is shown in *SI Appendix*, Table S2. Searches of peptides from individual, noncrosslinked NXF1 or Nsp1*^p^*^Bpa45^ yielded none of these crosslinked identifications.

### RNA FISH.

For detection of poly(A) RNA in cells, oligo-d(T) in situ hybridization was performed as we previously described (33). For detection of specific cellular mRNAs, the ViewRNA Cell Plus Assay Kit was used (Invitrogen, 88-19000-99). Additional experimental details are described in *SI Appendix*.

### Cell-Based Translation Assay (Including Statistical Analysis and Western Blot Conditions).

Fluc mRNA generation: To test the effect of Nsp1 on cellular translation, we expressed FLAG-tagged SARS-CoV-2 Nsp1 wildtype and indicated mutants in HEK293T cells and monitored translation of a cotransfected capped Firefly luciferase (Fluc) reporter mRNA. The Fluc mRNA was generated by in vitro transcription (IVT) using the mMESAGE mMACHINE T7 Transcription Kit (Invitrogen) and purified with the MEGAclear kit (Invitrogen) according to the manufacturer’s protocol. The template for the IVT was generated by PCR from the pCAG-Luc plasmid (Addgene, #55764) using the indicated primer sequences:

(T7-kozak-Fluc-Fw: 5′-TAATACGACTCACTATAGAGCCACCATGGAAGATGCCAAAAA-3′; PolyA-Fluc-Rv: 5′-TTTTTTTTTTTTTTTTTTTTTTTTTTTTTATTACACGGCGATCTTGCCG- 3′). The size and quality of the transcript were analyzed by agarose gel electrophoresis.

Translation Assay: HEK293T cells were cultured in 24-well plates and transfected with 500 ng pCl-neo vector or pCl-neo SARS-CoV-2 Nsp1 wildtype or mutants. Plasmid DNA was transfected using TransIT-LT1 Transfection Reagent (Mirus) in Opti-MEM Reduced Serum Medium (Gibco). The firefly luciferase (Fluc) mRNA (250 ng) was transfected 24 h after DNA transfection using Lipofectamine MessengerMAX (Invitrogen) in Opti-MEM Reduced Serum Medium (Gibco) according to the manufacturer’s recommendations. Prior to mRNA transfection, cells were washed once with fresh Dulbecco’s modified Eagle’s medium (Corning) supplemented with 10% FBS (Peak Serum) and penicillin/streptomycin (Corning). At 10 h after mRNA transfection, cells were lysed in Passive Lysis buffer (Promega) and the luminescence was quantified using Luciferase Assay System (Promega) according to the manufacturer’s instructions. Fluc values were normalized to the values of the empty vector samples. To determine statistical significance, a one-way ANOVA with multiple comparisons using Dunnett’s correction was performed. Nsp1 wildtype or Nsp1 mutant luciferase are compared to empty vector; *P-*value = 0.05. Western blot analysis is described in *SI Appendix*.

### In Vitro Translation Assay.

The in vitro translation assay was performed using the Reticulocyte Lysate IVT Kit (Ambion, Catalog number: AM1200) per the manufacturer’s instructions. Briefly, purified Nsp1 wild-type, Nsp1^D33K/E36K/E37K/E41K^, or GST proteins were incubated at a concentration of 1 μM with 250 ng of in vitro transcribed firefly Luciferase (Fluc) mRNA, reticulocyte lysate, and translation mix without methionine. Reactions were supplemented with unlabeled methionine (Promega, Catalog number: L118A) at a concentration of 50 μM. The samples were incubated at 30°C for 90 min. followed by incubation with 2.5 μL of 1 mg/mL RNase A (Qiagen, Catalog number: 19101) for 10 min. Samples were then stored on ice for immediate analysis. For analysis, samples were incubated with Passive Lysis Buffer (Promega, Catalog number: E1910) at a 1:10 dilution and the luminescence was quantified using the Luciferase Assay System (Promega, Catalog number: E1500) per the manufacturer’s instructions. To determine statistical significance, one-way ANOVA with multiple comparisons using Dunnett’s correction was performed. Nsp1 wild-type, Nsp1^D33K/E36K/E37K/E41K^, or GST were compared to control samples with no purified protein added.

### Metabolic Labeling with Click-iT HPG Alexa 594, FUNCAT Assay.

A549 cells were seeded at a density of 40,000/cells per well in a 24-well format on the top of round glass coverslips and transfected with 1 µg pCI-Neo-3xFLAG or pCl-neo-^3xFLAG^SARS-CoV-2-Nsp1 wildtype or mutants using Lipofectamine 3000 reagent in Opti-MEM Reduced Serum Medium. After 16 h of transfection, newly synthesized proteins were detected using the Click-iT™ HPG Alexa Fluor™ 594 Protein Synthesis Assay Kit. Additional details are described in *SI Appendix*.

### Generation of Recombinant Mutant SARS-CoV-2.

We used our previously described BAC-based SARS-CoV-2 reverse genetic systems (21) to generate the rSARS-CoV-2 containing the D33K/E36K/E37K/E41K mutation in the nsp1. Experimental details are described in *SI Appendix*. The P1 stock was aliquoted, titrated, deep sequenced (*SI Appendix*, Fig. S2), and stored at −80 °C until being used.

### PLA Combined with Immunofluorescence Microscopy.

A549-ACE2 cells were mock infected or infected with rSARS-CoV2^WT^ or rSARS-CoV2^D33K/E36K/E37K/E41K^. After 12 h, cells were fixed in 10 % formaldehyde in PBS at room temperature and permeabilized with 0.01% Triton X-100 in PBS for 10 min. Samples were then rinsed briefly with PBS and incubated with the Duolink blocking solution (Duolink In Situ Red Starter Mouse/Rabbit kit, DUO9210, Sigma-Aldrich Co. LLC) in a preheated humidity chamber for 30 min at 37 °C. Primary antibody cocktail [anti-NXF1 antibody, mouse monoclonal (T1076, Sigma-Aldrich Co. LLC, diluted at 1:250), and affinity purified rabbit anti-Nsp1 antibodies (3) (diluted at 1:100)] was diluted in the Duolink antibody diluent solution and added to the cells followed by incubation for 1 h at room temperature. Then the PLA was completed according to the manufacturer’s instructions. To identify infected cells, samples subjected to PLA were subsequently incubated overnight with affinity-purified rabbit anti-Nsp1 antibodies (3) at 4 °C. After washing with TBS-T, samples were incubated with the Alexa Fluor-488 goat anti-rabbit antibody (A110008, Molecular Probes, Life Technologies at 1:800 dilution). Next, samples were incubated with Hoechst 33342 (1.5 µg/mL, Molecular Probes) for nuclear counterstaining, then briefly washed with PBS for 5 min, and cells were kept in PBS. Infected cells were visualized using a FITC filter and PLA signals were recognized as red fluorescent spots using a TRITC filter. Antibody specificity was assessed in mock infected cells as PLA negative control. Fluorescence microscopy, image processing, and subsequent qualitative image analysis were performed as described in the ViewRNA methods section. Maximum intensity three-dimensional projection (MIP rendering) image processing was performed as we previously described (34).

### SARS-CoV-2 Growth Kinetics.

A549-ACE2 cells were seeded in 24-well format at a density of 100,000 cells per well. The next day, cells were infected at MOI 0.1 for 1h. Infections were performed in Viral Growth Media (VGM) (Dulbecco’s modified Eagle’s medium (Corning) supplemented with 2% FBS (Peak Serum), 1% nonessential amino acids (Gibco), 1% HEPES (Gibco), and 1% penicillin/streptomycin (Corning) at 37 °C and 5% CO_2_. The inoculum was removed and replaced with fresh infection media. Supernatants were collected at 12, 24, 36, and 48 hpi. Supernatants were stored at −80 °C before evaluation of viral titers. Titers were quantified by the plaque assay in Vero E6 cells as previously described (35). Additional details are described in *SI Appendix*.

### Immunofluorescence.

At the indicated time postinfection, cells were fixed with 4% formaldehyde overnight. Cells were permeabilized in 0.1% Triton in PBS and blocked in 3% BSA in PBS. Cells were then incubated in PBS containing 1% BSA and anti-SARS-CoV-2 N (1C7C7, Sigma Aldrich) at a 1:200 dilution for 1 h, at room temperature. Cells were washed in PBS three times for 5 min. Cells were incubated for 1 h at room temperature in PBS containing 1% BSA and 1:1,000 of AlexaFluor anti-mouse 488 and DAPI. Cells were washed in PBS three times and mounted in Antifade Gold mounting media. Images were acquired on the EVOS5000 microscope.

### SARS-CoV-2 Infection In Vivo.

Eight-week-old female B6.Cg-Tg(K18-ACE2)2Prlmn/J mice were obtained from The Jackson Laboratory and housed in a BSL-3 vivarium at the Icahn School of Medicine at Mount Sinai under the guidelines of the Institutional Animal Care and Use Committee (IACUC) of the Icahn School of Medicine at Mount Sinai (ISMMS). Experimental procedures are described in detail in *SI Appendix*.

## Supplementary Material

Appendix 01 (PDF)

## Data Availability

All study data are included in the article and/or *SI Appendix*.
