# Supplementary material for: Inhibition of mRNA nuclear export promotes SARS-CoV-2 pathogenesis
Source: Proc Natl Acad Sci U S A. 2024 May 20;121(22):e2314166121. doi: 10.1073/pnas.2314166121 (PMC11145185; doi:10.1073/pnas.2314166121)
Supplement: Supplementary file 1 — Appendix 01 (PDF) [file pnas.2314166121.sapp.pdf]

Supplementary Information

Inhibition of mRNA Nuclear Export Promotes SARS-CoV-2 Pathogenesis

Menghan Mei[1]*, Anastasija Cupic[2, 3]*, Lisa Miorin[2,4], Chengjin Ye[5], Tolga Cagatay[6], Ke Zhang[6,7], Komal Patel[1,8], Natalie Wilson[1], W Hayes McDonald[1,9], Nicholas A. Crossland[10,11], Ming Lo[10], Magdalena Rutkowska[2,3], Sadaf Aslam[2], Ignacio Mena[2,4¶], Luis Martinez-Sobrido[5], Yi Ren[1§], Adolfo García-Sastre[2,4,12,13,14§], and Beatriz M. A. Fontoura[6§]

[1]Department of Biochemistry, Center for Structural Biology, Vanderbilt University School of Medicine, Nashville, TN 37232, USA

[2]Department of Microbiology, Icahn School of Medicine at Mount Sinai, New York, NY 10029, USA

[3]Graduate School of Biomedical Sciences, Icahn School of Medicine at Mount Sinai, New York, NY 10029, USA

[4]Global Health and Emerging Pathogens Institute, Icahn School of Medicine at Mount Sinai, New York, NY 10029, USA

[5]Texas Biomedical Research Institute, San Antonio, TX 78227, USA

[6]Department of Cell Biology, University of Texas Southwestern Medical Center, Dallas, Texas 75390, USA

[7]Key Laboratory of Molecular Virology and Immunology, Chinese Academy of Sciences, Shanghai 200031, China.

[8]Arpirnaut Program, Vanderbilt University School of Medicine, Nashville, TN 37232, USA

[9]Mass Spectrometry Research Center, Vanderbilt University School of Medicine, Nashville, TN 37232, USA

[10]Comparative Pathology Laboratory, National Emerging Infectious Diseases Laboratories, Boston University, Boston, MA 02215, USA

[11]Department of Pathology and Laboratory Medicine, Boston University School of Medicine, Boston, MA 02118, USA

[12]Department of Medicine, Division of Infectious Diseases, Icahn School of Medicine at Mount Sinai, New York, NY 10029, USA

[13]Department of Pathology, Molecular and Cell-Based Medicine, Icahn School of Medicine at Mount Sinai, New York, NY 10029, USA

[14]The Tisch Cancer Institute, Icahn School of Medicine at Mount Sinai, New York, NY 10029, USA

*These authors equally contributed to the manuscript.

¶Ignacio Mena's current address: The Scripps Research Institute.10550 North Torrey Pines Road, La Jolla, CA 92037

§Corresponding Authors: Yi Ren (yi.ren@vanderbilt.edu), Adolfo García-Sastre (adolfo.garcia-sastre@mssm.edu), and Beatriz M. A. Fontoura (Beatriz.Fontoura@UTSouthwestern.edu).

This pdf file includes:

Figures S1-S5

Tables S1-S3

Materials and Methods

References

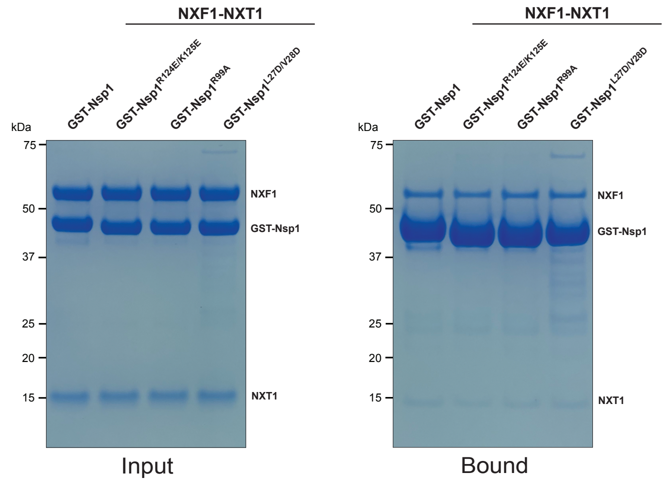

Fig. S1: Previously characterized Nsp1-N residues implicated in ribosome binding, RNA degradation, or DNA polymerase α-primase binding do not disrupt NXF1-NXT1 binding. *In vitro* GST pull-down assays were performed with GST-tagged Nsp1 variants and NXF1-NXT1.

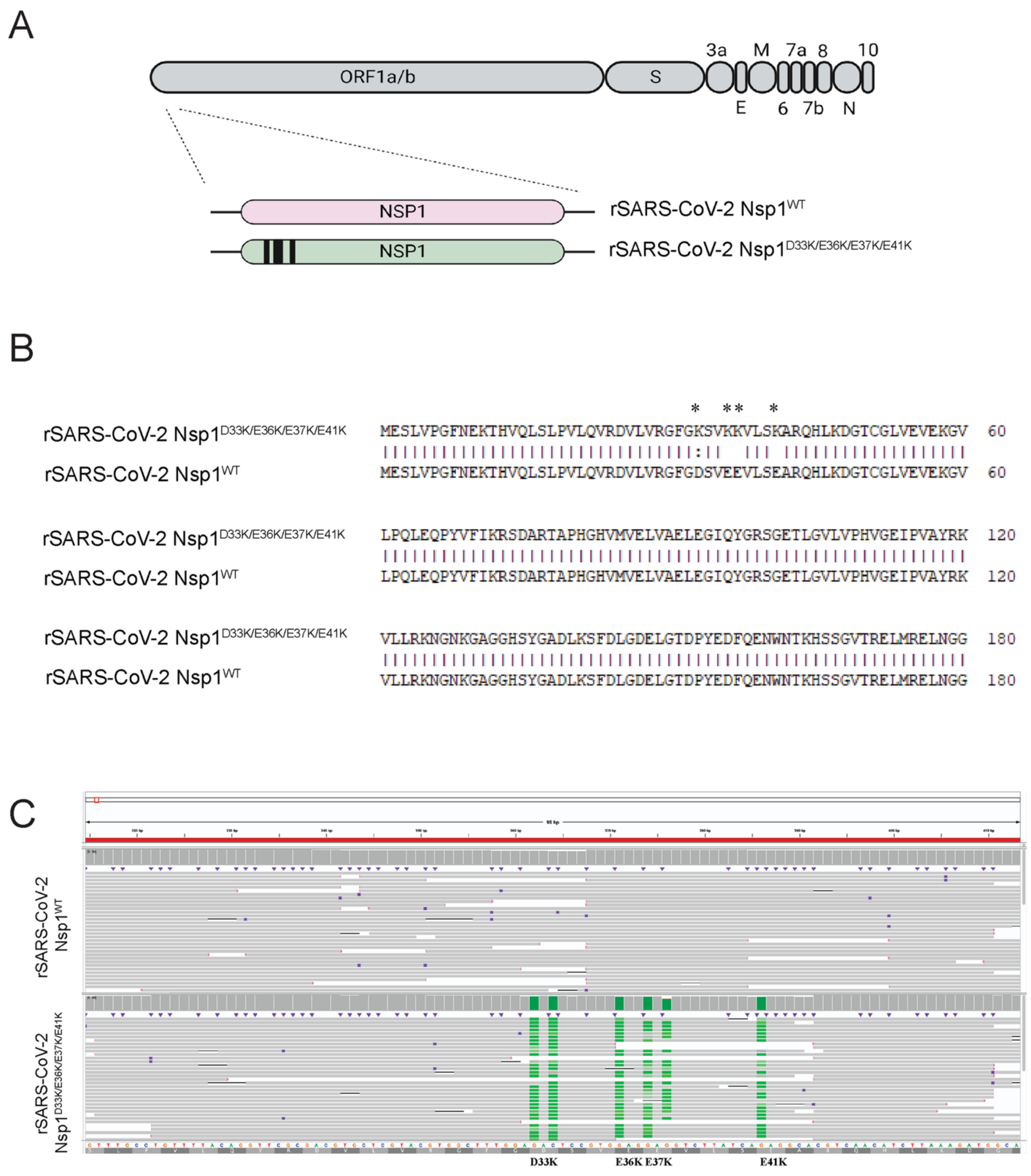

Fig. S2: Sequencing results of rSARS-CoV-2 Nsp1^{WT} and rSARS-CoV-2 Nsp1$^{D33K/E36K/E37K/E41K}$ virus stock. *(A)* Schematic illustration of the genome organization of rSARS-CoV-2 Nsp1^{WT} and Nsp1$^{D33K/E36K/E37K/E41K}$ viruses used in our studies. *(B)* Alignment of the rSARS-CoV-2 Nsp1$^{D33K/E36K/E37K/E41K}$ virus stock amplicon to the SARS-CoV-2 Nsp1 reference

sequence. *(C)* Alignment of the MinIon sequencing reads for plaque purified rSARS-CoV-2 $NSP1^{WT}$ and rSARS-CoV-2 $Nsp1^{D33K/E36K/E37K/E41K}$ to the SARS-CoV-2 WA1/2020 reference sequence.

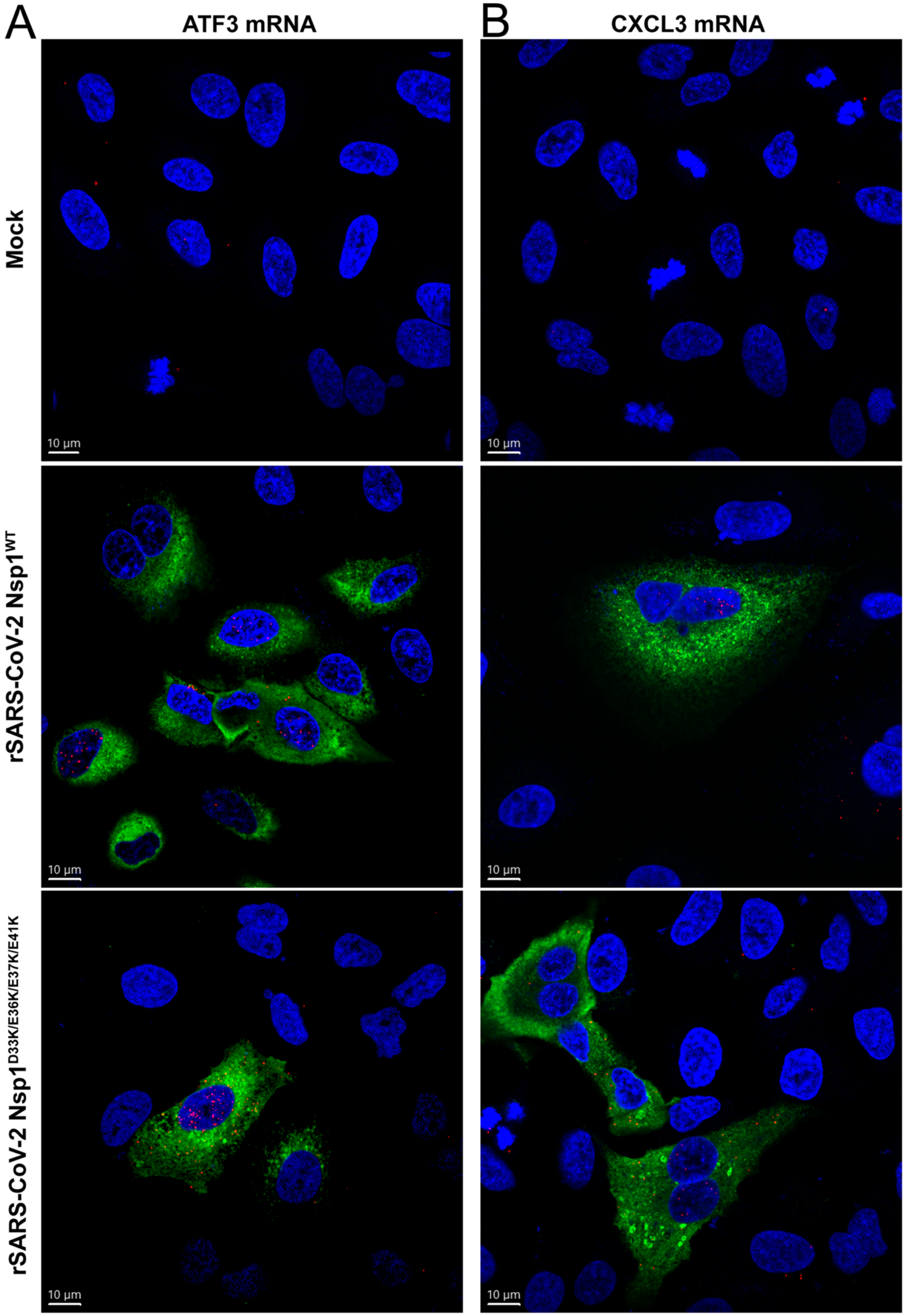
A
ATF3 mRNA
B
CXCL3 mRNA
Mock
rSARS-CoV-2 Nsp1WT
rSARS-CoV-2 Nsp1D33K/E36K/E37K/E41K
10 µm

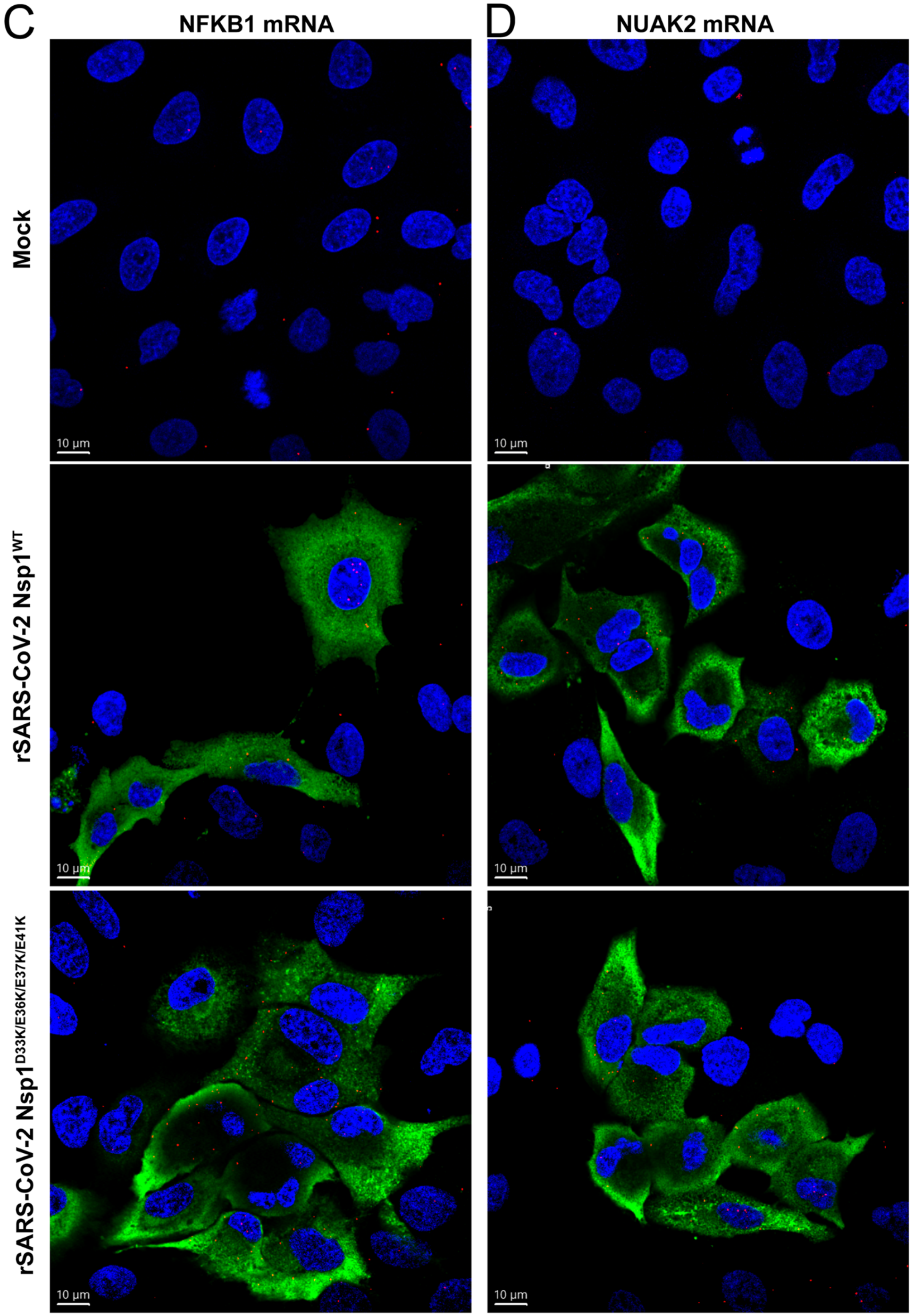

C
NFKB1 mRNA
D
NUAK2 mRNA
Mock
rSARS-CoV-2 Nsp1WT
rSARS-CoV-2 Nsp1D33K/E36K/E37K/E41K
10 µm

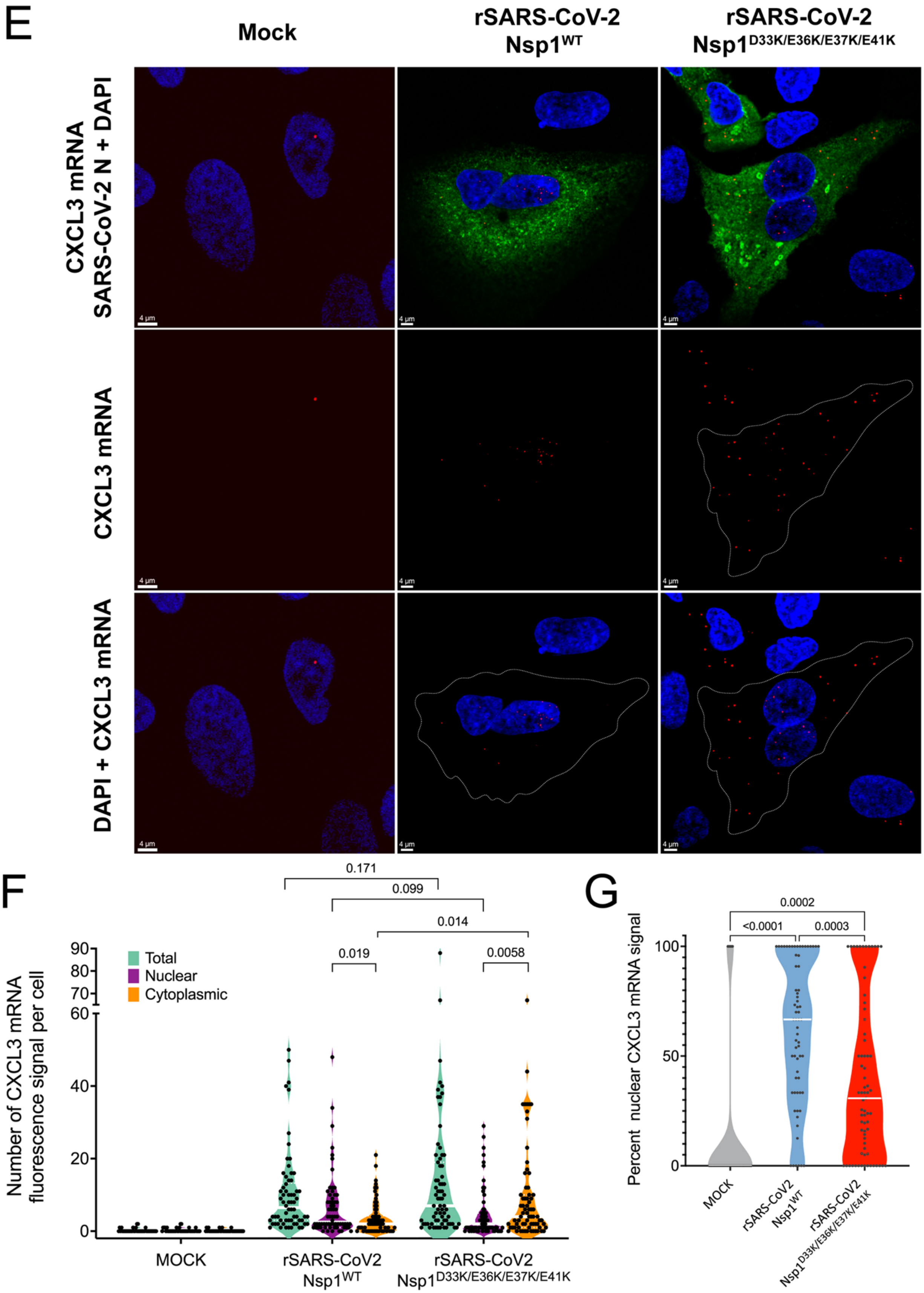

E
Mock
rSARS-CoV-2 Nsp1WT
rSARS-CoV-2 Nsp1D33K/E36K/E37K/E41K
CXCL3 mRNA SARS-CoV-2 N + DAPI
CXCL3 mRNA
DAPI + CXCL3 mRNA
4 µm
F
Total
Nuclear
Cytoplasmic
Number of CXCL3 mRNA fluorescence signal per cell
0.171
0.099
0.014
0.019
0.0058
MOCK
rSARS-CoV2 Nsp1WT
rSARS-CoV2 Nsp1D33K/E36K/E37K/E41K
G
Percent nuclear CXCL3 mRNA signal
0.0002
<0.0001
0.0003
MOCK
rSARS-CoV2 Nsp1WT
rSARS-CoV2 Nsp1D33K/E36K/E37K/E41K

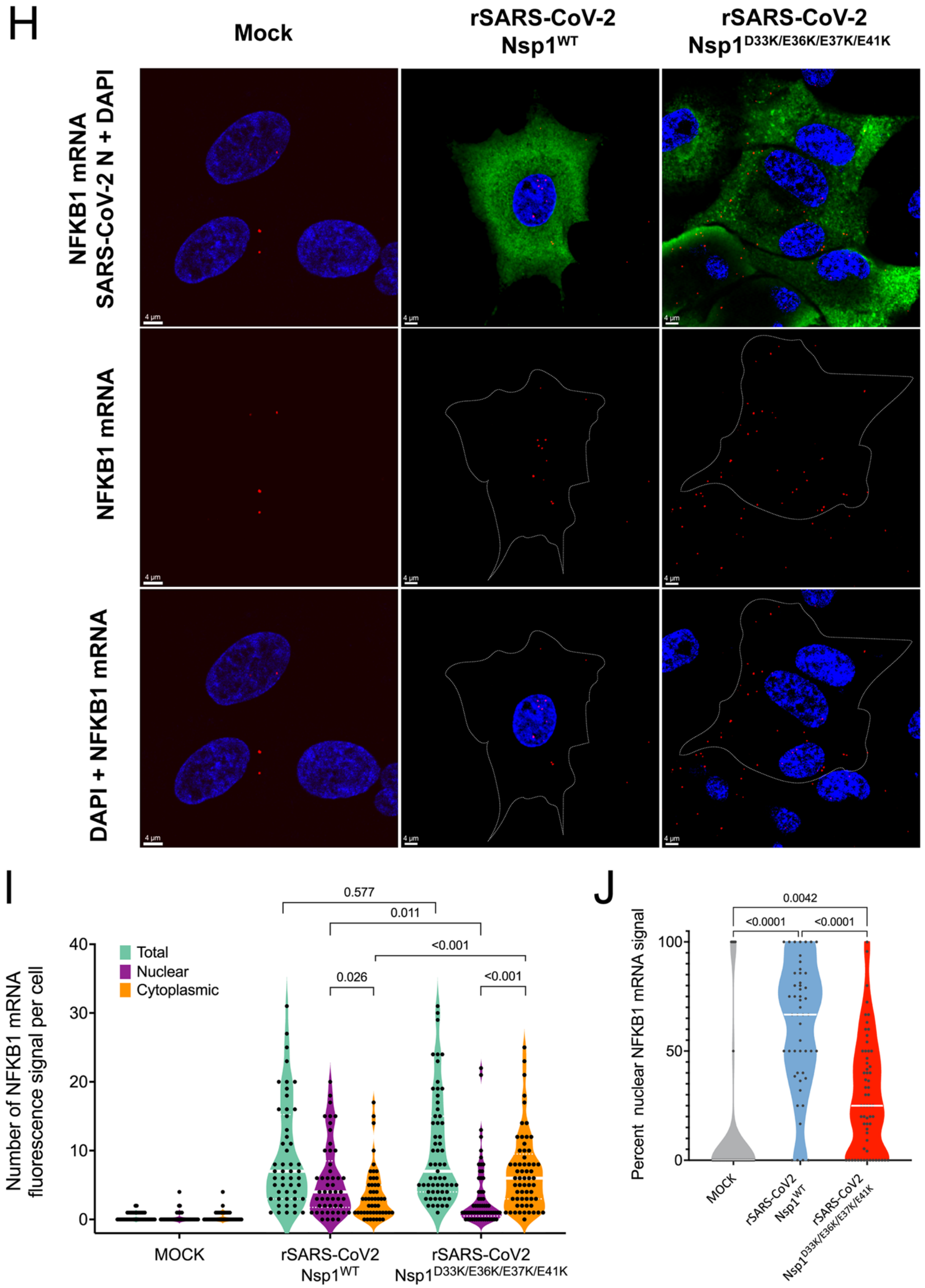
H
Mock
rSARS-CoV-2
Nsp1WT
rSARS-CoV-2
Nsp1D33K/E36K/E37K/E41K
NFKB1 mRNA
SARS-CoV-2 N + DAPI
NFKB1 mRNA
DAPI + NFKB1 mRNA
4 µm
I
0.577
0.011
<0.001
0.026
<0.001
Total
Nuclear
Cytoplasmic
Number of NFKB1 mRNA fluorescence signal per cell
0
10
20
30
40
MOCK
rSARS-CoV2
Nsp1WT
rSARS-CoV2
Nsp1D33K/E36K/E37K/E41K
J
0.0042
<0.0001
<0.0001
Percent nuclear NFKB1 mRNA signal
0
50
100
MOCK
rSARS-CoV2
Nsp1WT
rSARS-CoV2
Nsp1D33K/E36K/E37K/E41K

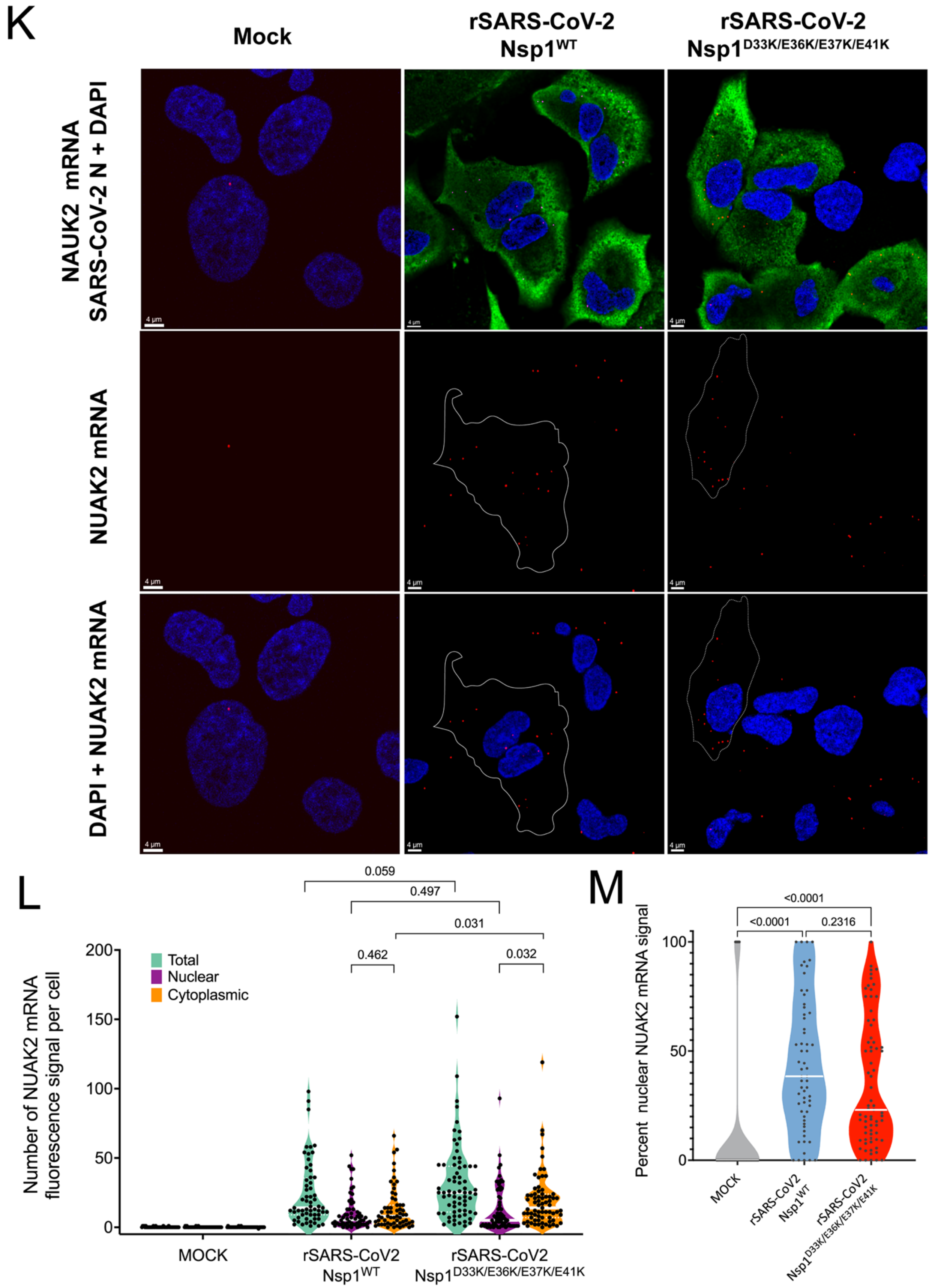

K
Mock
rSARS-CoV-2
Nsp1WT
rSARS-CoV-2
Nsp1D33K/E36K/E37K/E41K
NAUK2 mRNA
SARS-CoV-2 N + DAPI
NUAK2 mRNA
DAPI + NUAK2 mRNA
4 µm
L
Number of NUAK2 mRNA fluorescence signal per cell
Total
Nuclear
Cytoplasmic
0.059
0.497
0.031
0.462
0.032
200
150
100
50
0
MOCK
rSARS-CoV2
Nsp1WT
rSARS-CoV2
Nsp1D33K/E36K/E37K/E41K
M
Percent nuclear NUAK2 mRNA signal
<0.0001
<0.0001
0.2316
100
50
0
MOCK
rSARS-CoV2
Nsp1WT
rSARS-CoV2
Nsp1D33K/E36K/E37K/E41K

Fig. S3: SARS-CoV-2 Nsp1$^{D33K/E36K/E37K/E41K}$ is unable to inhibit cellular mRNA export. *(A-D)* A549-ACE2 cells were infected with rSARS-CoV-2 Nsp1^{WT} or SARS-CoV-2 Nsp1$^{D33K/E36K/E37K/E41K}$ at MOI 0.25 for 24 h. Cells were *subjected* to the ViewRNA Cell Plus Assay to detect specific cellular mRNAs, as depicted in the figure, and immunofluorescence microscopy to detect SARS-CoC-2 N protein (green). Nuclei is stained in blue with DAPI. Images show the original field of view (at 63x magnification). *(A)* ATF3 mRNA, *(B)* CXCL3 mRNA, *(C)* NFKB1 mRNA, and *(D)* NUAK2 mRNA. Images in *(E)*, *(H)* and *(K)* correspond to high magnification areas from *(B-D)*, and *(A)* is in the main figure. *(F,I and L)* Scatter plot representations of single cell mRNA [*(F)* CXCL3, *(I)* NFKB1, and *(L)* NAUK2)] absolute fluorescence signal in whole cell (total), nucleus, or cytoplasm of mock, rSARS-CoV-2 Nsp1^{WT} or rSARS-CoV-2 Nsp1$^{D33K/E36K/E37K/E41K}$ infected cells. Each dot represents a cell. The dashed lines represent quartiles and the white line represents median value. *(G, J and M)* The calculated percent nuclear mRNA signal is presented as scattered plot for each individual cell. CXCL3 mRNA (mock n= 42 cells; wild-type virus n= 66 cells; mutant virus n= 71 cells), NFKB1 mRNA (mock n= 48 cells; wild-type virus n= 50 cells; mutant virus n= 61 11 cells), NUAK2 mRNA (mock n= 49 cells; wild-type virus n= 60 cells; mutant virus n= 71 cells (mock n= 25 cells; wild-type virus n= 51 cells; mutant virus n= 51 cells). Each dot represents a cell and the median value is depicted by a white line. Statistical analysis was performed using one-way ANOVA with a Tukey post test and p values are depicted in the figure.

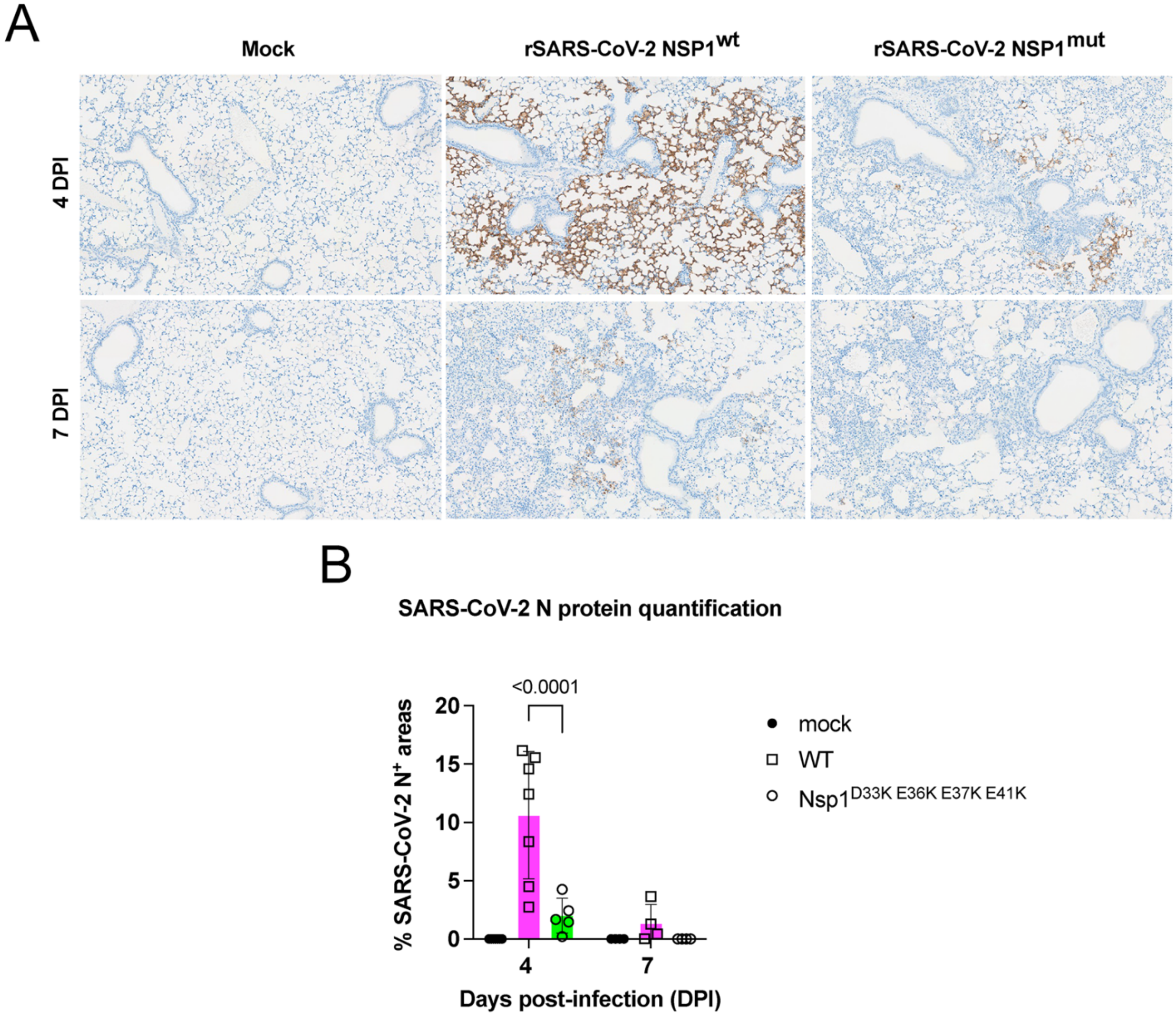

Fig. S4: Reduced SARS-CoV-2 N antigen load in the lungs of K18hACE2 mice infected with rSARS-CoV-2 Nsp1$^{D33K/E36K/E37K/E41K}$ compared to rSARS-CoV-2^{WT}. Representative images of lung tissues collected from each virally infected cohort at the indicated time points and subjected to IHC with SARS-CoV-1/2 N antibody. Viral antigen load within permissive alveolar type I and II pneumocytes was observed at both time points, with little to no detectable antigen at 7 DPI in the rSARS-CoV-2 Nsp1$^{D33K/E36K/E37K/E41K}$ cohort. The graph illustrates quantification analysis of the whole slide positive pixel area.

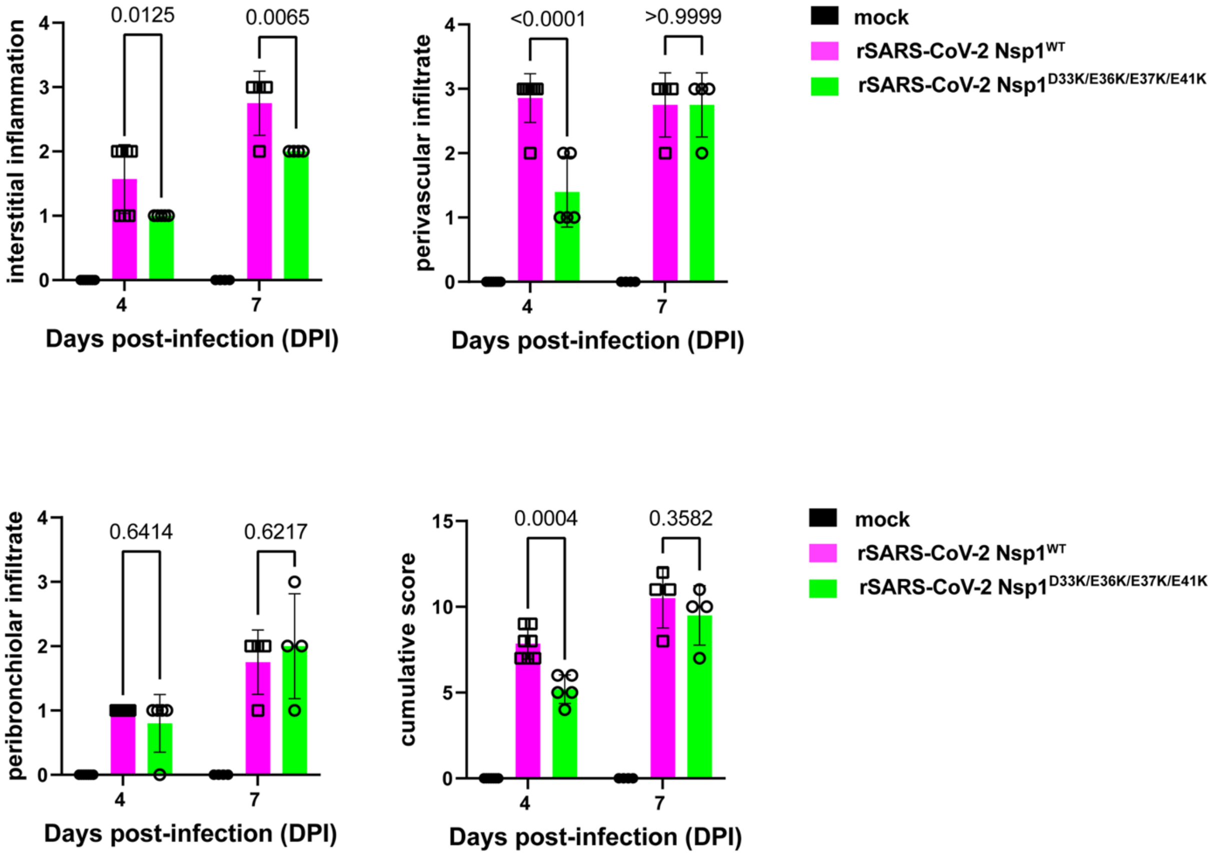

Fig. S5: Histology scores related to figure 8e. Inflammatory cell infiltrates in each anatomical compartment (peribronchiolar, perivascular, and interstitium) of SARS-CoV-2^{WT} or rSARS-CoV-2 Nsp1$^{D33K/E36K/E37K/E41K}$ infected K18-hACE2 mice as well as overall percentage of pulmonary parenchyma affected were scored using an ordinal scoring system. Values were compared using two-way ANOVA with Tukey's correction. *p* values are shown.

Table S1. Summary of binding of Nsp1 variants to NXF1-NXT1

SARS-CoV-2 Nsp1	Characteristics/Functions	NXF1-NXT1 binding
Wild type	-	***
1-129	Nsp1-N	*
133-180	Nsp1-C	*
**Mutations on Nsp1-N**		
L27D/V28D	Hydrophobic (Pol α-primase binding)	***
D33K/E36K/E37K/E41K	Acidic	*
E55A/E57A/K58E	Acidic/Basic	***
H81E/H83E	Basic	***
E91K/L92A/E93K/I95A	Acidic/Hydrophobic	***
R99A	Basic (ribosome binding)	***
R124E/K125E	Basic (mRNA cleavage, ribosome binding)	***
K129D	Basic	***

Table S2. Analysis of Nsp1^{pBPa45} crosslinks to NXF1

Nsp1^{pBPa45} peptide[a]	NXF1 peptide	Site of *p*Bpa crosslink[b]	Spectral Count
^{44}[QxLK]^{47}	^{187}[ISIIINSSAPPHTILNELKPEQVEQLK]^{213}	**I190 (7)**, I189 (2), P196 (2), S194 (1),	12
^{44}[QxLK]^{47}	^{159}[AQFFVEDASTASALKAVNYK]^{178}	V163 (1), D165 (1), D166 (1), T168 (1)	4
^{44}[QxLK]^{47}	^{143}[CSVPFTPIEFHYENTRAQFFVEDASTASALK]^{173}	P146 (1), F161 (1), E164 (1)	3
^{44}[QxLK]^{47}	^{133}[AWLLSMIQSK]^{142}	A133 (1), L136 (1)	2

[a]X=*p*Bpa
[b]Number in parenthesis for indicated site of crosslink gives spectral count for that species.

Table S3

**Ordinal Histopathology Criteria**	
% of total examined pulmonary parenchyma affected by histopathologic lesions attributable to SARS-CoV-2 infection	
0	Absent
1	Minimal, >0%-10%
2	Mild, >10%-30%
3	Moderate, 30-80%
4	Severe, >80%-100%
**Interstitium:**	
0	Within normal limits
1	Mild focal to multifocal mononuclear +/- neutrophil interstitial infiltrate
2	Moderate focal to multifocal mononuclear +/- neutrophil interstitial infiltrate with +/- increased interstitial macrophages +/- type 2 pneumocyte hypertrophy
**Blood vessels**	
0	Within normal limits
1	Mild focal to multifocal perivascular mononuclear infiltrates forming loose cuffs (1-5 cell thick) of medium-caliber vessels and small-caliber with +/- endothelial hypertrophy +/- neutrophils
2	Moderate focal to multifocal, dense cuffs of perivascular mononuclear infiltrates of small-caliber and medium-caliber vessels that are >5- 10 cell thick, with moderate to prominent endothelial hypertrophy +/- neutrophils; the mononuclear infiltrates extend to the interstitium of adjacent alveolar septa
**Airways cell death (bronchioles)**	
0	Within normal limits, rare, rounded degenerate bronchiolar epithelial cells may present
**Peribronchiolar infiltrates**	

0	Within normal limits, a trace number of individual mononuclear infiltrates may present
1	Mild peribronchiolar mononuclear infiltrates
**Pulmonary hemorrhage**	
0	Absent
1	Mild, focal to multifocal hemorrhage obscures alveolar spaces

Materials and Methods

Plasmids

Human NXF1 (UniProtKB accession number Q9UBU9, residues 117-619), NXF1 mutants, SARS-CoV-2 Nsp1 (UniProtKB accession number P0DTD1, residues 1-180), Nsp1-N (residues 1-129), Nsp1-C (residues 133-180), and Nsp1 mutants (*p*Bpa containing and non *p*Bpa containing) were cloned into BamHI and NotI sites of a pGEX-4T-1 vector modified to contain a TEV-cleavable N-terminal GST tag. The construct of Nsp1 (residues 1-180) in the pGEX-6P-1 vector was described previously (1). Full-length human NXT1 (UniProtKB accession number Q9UKK6, residues 1-140) was cloned into NdeI and EcoRI sites of the pBB75 vector to be co-expressed with NXF1. All 3xFlag tagged Nsp1 expression constructs used for transient transfections were cloned into the pCINeo-3XFlag mammalian expression vector using the NEBuilder HiFi DNA Assembly Cloning Kit according to the manufacturer's instructions (New England BioLabs, MA). pCAG-Luciferase was a gift from Snorri Thorgeirsson (Addgene plasmid # 55764). All expression vectors were sequenced for verification.

Antibodies

Anti-Flag M2 monoclonal antibody was used at 1:1000 dilution (Sigma-Aldrich, F1804), Alexa Fluor 488–conjugated donkey anti-mouse IgG antibody was used at 1:1000 dilution (Invitrogen), Hoechst 33258 (Thermo Fisher Scientific, H3569), Monoclonal ANTI-FLAG M2-peroxidase (HRP) (Sigma-Aldrich, A8592), β-Tubulin (9F3) Rabbit mAb (HRP conjugate) (Cell Signaling Technology, 5346S). Anti- SARS-CoV-1/2 N monoclonal antibody, clone 1C7C7 (Sigma Aldrich; Catalog number: ZMS1075-4X25UL). Goat Anti-Mouse IgG (H+L) AlexaFluor488 (Invitrogen; Catalog number: A21202). Rabbit polyclonal anti-Nsp1 antibodies were generated as previously described (1) and affinity purified as reported (2).

Protein purification

GST-NXF1$^{117-619}$-NXT1 wild-type and mutants were co-expressed in *E. coli* Rosetta cells (EMD Millipore, 70954). Protein expression was induced at an optical density at 600 nm of 0.6 with 0.5 mM IPTG at 18 °C and cells were incubated for 16 h. Cells were collected and lysed with a cell disruptor (Avestin) in a wash buffer (50 mM Tris, pH 8.0, 500 mM NaCl, 1 mM DTT) supplemented with a protease inhibitor mix containing 1 mM PMSF, 0.1 mM AEBSF, and 5 mg/L aprotinin. Cleared cell lysate was incubated with Glutathione Sepharose resins (Cytiva, 17075605), which were washed extensively with the wash buffer. Bound proteins were eluted from the Glutathione Sepharose resins with the wash buffer supplemented with 10 mM reduced glutathione, followed by digestion with GST tagged TEV protease (GST-TEV) at 4 °C. NXF1$^{117-619}$-NXT1 were applied to a HiTrap SP column (Cytiva) and eluted with a 150-400 mM NaCl gradient in a buffer containing 10 mM HEPES, pH 7.0, 1 mM DTT. HiTrap elution was passed through Glutathione Sepharose resins to remove undigested sample. NXF1$^{117-619}$-NXT1 were further purified using a Superdex 200 column (Cytiva) equilibrated with 10 mM tris, pH 8.0, 300 mM NaCl, and 0.5 mM TCEP. GST-TEV digested NXF1$^{117-619}$-NXT1 mutants were buffer exchanged to a buffer containing 50 mM Tris, pH 8.0, 500 mM NaCl, 1 mM DTT and passed through Glutathione Sepharose resins to remove GST and undigested sample. NXF1$^{117-619}$-NXT1 mutants were further purified using a Superdex 200 column equilibrated with 10 mM tris, pH 8.0, 300 mM NaCl, and 0.5 mM TCEP.

GST-Nsp1 and GST-Nsp1 variants (non *p*Bpa-containing) were expressed in *E. coli* Rosetta cells. Protein expression was induced at an optical density at 600 nm of 0.6 with 0.5 mM IPTG at 18 °C and cells were incubated for 16 h. GST-Nsp1 and GST-Nsp1 variants were first purified by glutathione-affinity chromatography, followed by a Superdex 200 column equilibrated with 10 mM tris, pH 8.0, 300 mM NaCl, and 0.5 mM TCEP. For the purification of untagged Nsp1 and Nsp1$^{D33K/E36K/E37K/E41K}$, glutathione-affinity purified GST-Nsp1 and GST-Nsp1$^{D33K/E36K/E37K/E41K}$ were

applied to a Source 15Q column (Cytiva) and eluted with a 75 mM to 450 mM NaCl gradient. GST-Nsp1 Source 15Q elution was treated with GST-tagged PreScission Protease to remove the GST tag. GST-Nsp1$^{D33K/E36K/E37K/E41K}$ Source 15Q elution was treated with GST-TEV to remove the GST-tag. Digested Nsp1 and Nsp1$^{D33K/E36K/E37K/E41K}$ were passed through Glutathione Sepharose resins to remove GST and undigested protein. Nsp1 and Nsp1$^{D33K/E36K/E37K/E41K}$ were further purified using a Superdex 75 column (Cytiva) equilibrated with 10 mM tris, pH 8.0, 300 mM NaCl, and 0.5 mM TCEP.

GST-Nsp1^{pBpa45} was co-expressed with a tRNA synthetase/tRNA pair encoded by a plasmid pEVOL-pBpF (Addgene, 31190) in *E. coli* BL21 (DE3) (NEB, C2527). Arabinose was added to the cell culture to 0.02% (w/v) at an optical density at 600 nm of 0.3. Cells were spun down at an optical density at 600 nm of 0.6 and resuspended in 5-fold less volume media in the presence of 1 mM *p*Bpa (AEchem) and 0.02% (w/v) arabinose. Cells were incubated at 16 °C for 1h and protein expression was then induced with 0.5 mM IPTG at 16 °C for 16h. Nsp1^{pBpa45} was first purified by glutathione-affinity chromatography, followed by digestion with GST-TEV at 4 °C. Sample was buffer exchanged to a buffer containing 50 mM Tris, pH 8.0, 500 mM NaCl, 1 mM DTT and passed through Glutathione Sepharose resins to remove GST and undigested sample. Nsp1^{pBpa45} was further purified using a Superdex 200 column equilibrated with 10 mM tris, pH 8.0, 300 mM NaCl, and 0.5 mM TCEP. All purified proteins were concentrated and stored at −80°C.

RNA fluorescence *in situ* hybridization (FISH)

For detection of poly(A) RNA cells, oligo-d(T) *in situ* hybridization was performed as we previously described (3). Images were acquired with a Zeiss Axiovert 200 M automated microscope using a 63X Plan-APOCHROMAT lens (1.4 numerical aperture) and captured by a Hamamatsu ORCA-spark CMOS monochrome camera. Images were recorded sequentially for the antibody, RNA

and DNA fluorescence channels respectively using a step size of 0.5 μm (total z size 10 μm). Acquired z stack images were blind deconvolved using Autoquant X (Media Cybernetics).

For detection of specific cellular mRNAs, ViewRNA Cell Plus Assay Kit was used (Invitrogen, 88-19000-99). Probes used: ViewRNA Type 6 Probe Set (Invitrogen,ThermoFisher), ATF3 (Assay ID: VA6-3168675-VCP; Catalog #: VX-01; Detection Label: Alexa Fluor 647), CXCL3 (Assay ID: VA6-3168903-VCP; Catalog #: VX-01; Detection Label: Alexa Fluor 647), NUAK2 (Assay ID: VA6-3180987-VCP; Catalog #: VX-01; Detection Label: Alexa Fluor 647), NFKB1 (Assay ID: VA6-16931-VCP, Catalog #: VX-01, Detection Label: Alexa Fluor 647). A549-ACE2 cells were seeded at a density of 20,000/cells per well in a 24-well format on top of round glass coverslips. Cells were seeded in culture media (Dulbecco's modified Eagle's medium (Corning) supplemented with 10% FBS (Peak Serum), and 1% penicillin/streptomycin (Corning) and incubated at 37 °C and 5% CO_2. The next day, A549-ACE2 cells were infected with recombinant SARS-CoV-2 WT or rSARS-CoV-2 Nsp1 D33K/E36K/E37K/E41K at an MOI of 0.25. Virus growth media was used for the infections (Dulbecco's modified Eagle's medium (Corning) supplemented with 2% FBS (Peak Serum), 1% non-essential amino acids (Gibco), 1% HEPES (Gibco) and 1% penicillin/streptomycin (Corning). The infections were performed under BSL3 containment in accordance with the biosafety protocols developed by the Icahn School of Medicine at Mount Sinai. After 24 hours of infection, cells were fixed according to the manufacturer's instructions. The manufacturer's protocol for samples processed on coverslips in a 24-well plate was followed with additional modifications: A hybridization oven (HybEZ™ II Hybridization System, ACD) at 40 ^{0}C was used for all steps requiring 40 ^{0}C. A humidified staining tray (HybEZ™ II Hybridization System, ACD) was used for all steps requiring a humidified staining tray. Primary antibody was a mouse monoclonal anti-SARS-CoV1/2-N antibody (clone 1C7C7) and was used at a dilution of 1:300. Probes were used at a 1:100 dilution. Incubations were lengthened for steps 14 and beyond. Step 18 was a 2.5 h incubation. Step 26 was a 1.5 h incubation. Step 29 was a 1.5 h

incubation. Step 31 was a 1.5 h incubation. Antifade Gold mounting media with DAPI was used for mounting. Glass slides from Matsunami (ref: SUMGP15) were used in these experiments. Coverslips were sealed with clear nail polish after mounting. Samples were stored at 4 ^{0}C in the dark before acquisition. Images were acquired the day after staining to avoid fading of the probe. Images were acquired by confocal microscopy using the LSM-880 microscope. The image acquisition settings are 0.5 µm optical z-sections spanning a cell volume with a 63x oil objective.

Imaris-assisted image analysis for quantitative FISH

For quantification of poly(A) RNA, the Imaris software package Cells module (Bitplane, Version 9.8.2) was used for segmentation and signal analysis within the cytoplasm and nucleus. First, nuclei (Hoechst 33258) were segmented using an automated threshold (based on the intensity distribution histogram) in 405 nm laser line. Nuclei populations were filtered to remove large nuclei aggregates (upper nuclei volume threshold and lower threshold to manually remove fragments of nuclei at the edges of the stack) using the Imaris surface tool. Next, 3xFlag-tagged Nsp1 (Alexa 488 secondary) and total cellular poly(A) RNA signal (Cy5-poly(A) probe) were identified at 488 nm and 633 nm, respectively, using the Imaris surface tool. For objective cell body identification, high intensity and exposure thresholds were set for both 488 and 630 channels. The single cell distribution of poly(A) RNA signal was extracted and calculated as the intensity sum of voxels within the detected total cell volume or nuclear volume using the masking and statistic tools of Imaris software package Cells module. Brightness and contrast were linearly enhanced using Adobe Photoshop's Level tool. Statistical analysis was carried out using the one-way ANOVA with a Tukey post test (Prism 9, GraphPad).

For quantification of specific cellular mRNAs, the Imaris software package Cells module (Bitplane, Version 9.8.2) was used to identify and create a conventional 2-dimensional maximum intensity image of the nucleus (chromatin), SARS-CoV2 N and ViewRNA ISH signal. First, nuclei (DAPI)

were segmented using an automated threshold (based on the intensity distribution histogram) in 405 nm laser line. Identified nuclei populations were filtered to remove large nuclei aggregates (upper nuclei volume threshold and lower threshold to manual remove fragments of nuclei at the edges of the stack) using the Imaris surface tool. Next, SARS-CoV2 N expression (protein marker for infected cells) and ViewRNA ISH signal for selected mRNA were identified in 488 nm laser line using Imaris surface tool and 467 nm laser line using the Imaris spot tool, respectively. Cell segmentation was performed in manual drawing mode at 1 mm vertex spacing. The seed spot size used was 0.2-0.7 mm. For objective mRNA foci center identification, automatic thresholds were used to filter raw spot quality. After identification of all mRNA spots in the cell, the nuclear mRNA spots were segmented, setting spot filter function to the “shortest distance to Nucleus”, upper-threshold to 0, and turning the lower-threshold off. Brightness and contrast were linearly enhanced using Adobe Photoshop's Level tool. Statistical analysis was carried out using the one - way ANOVA with a Tukey post test (Prism 9, GraphPad).

Cell-based translation assay (including statistical analysis and Western blot conditions)

Fluc mRNA generation: to test the effect of Nsp1 on cellular translation we expressed FLAG-tagged SARS-CoV-2 Nsp1 wildtype and indicated mutants in HEK293T cells and monitored translation of a co-transfected capped Firefly luciferase (Fluc) reporter mRNA. The Fluc mRNA was generated by *in vitro* transcription (IVT) using the mMESAGE mMACHINE T7 Transcription Kit (Invitrogen) and purified with the MEGAclear kit (Invitrogen) according to the manufacturer’s protocol. The template for the IVT was generated by PCR from the pCAG-Luc plasmid (Addgene, #55764) using the indicated primer sequences:

(T7-kozak-Fluc-Fw: 5’-TAATACGACTCACTATAGAGCCACCATGGAAGATGCCAAAAA-3’;

PolyA-Fluc-Rv: 5’-TTTTTTTTTTTTTTTTTTTTTTTTTTTTTTATTACACGGCGATCTTGCCG- 3’).

The size and quality of the transcript were analyzed by agarose gel electrophoresis.

Translation Assay: HEK293T cells were cultured in 24-well plates and transfected with 500ng pCI-neo vector or pCI-neo SARS-CoV-2 Nsp1 wildtype or mutants. Plasmid DNA was transfected using TransIT-LT1 Transfection Reagent (Mirus) in Opti-MEM Reduced Serum Medium (Gibco). The firefly luciferase (Fluc) mRNA (250 ng) was transfected 24 hours after DNA transfection using Lipofectamine MessengerMAX (Invitrogen) in Opti-MEM Reduced Serum Medium (Gibco) according to the manufacturer's recommendations. Prior to mRNA transfection, cells were washed once with fresh Dulbecco's modified Eagle's medium (Corning) supplemented with 10% FBS (Peak Serum) and penicillin/streptomycin (Corning). At 10 hours after mRNA transfection, cells were lysed in Passive Lysis buffer (Promega) and the luminescence was quantified using Luciferase Assay System (Promega) according to the manufacturer's instructions. Fluc values were normalized to the values of the empty vector samples. To determine statistical significance, a one-way ANOVA with multiple comparisons using Dunnett's correction was performed. Nsp1 wildtype or Nsp1 mutant luciferase are compared to empty vector; p-value = 0.05.

Western Blot: cell lysates from the translation assay were resuspended in 2X Laemmli sample buffer (Bio-Rad Laboratories) and boiled prior to loading. Samples were run on a 4-20% gradient polyacrylamide gel and transferred onto polyvinylidene fluoride (PVDF) membranes (Bio-Rad Laboratories) using the Trans-Blot Turbo Transfer System (Bio-Rad Laboratories). Membranes were blocked in Tris-buffered saline containing 0.1% Tween 20 detergent (TBS-t) with 5% nonfat dry milk. Primary antibodies were used at dilutions of 1:1000 in 3% bovine serum albumin in TBS-T.

Metabolic labeling with Click-iT HPG Alexa 594, Fluorescent non-canonical amino acid tagging (FUNCAT) assay

A549 cells were seeded at a density of 40,000/cells per well in a 24-well format on the top of round glass coverslips and transfected with 1µg pCI-Neo-3xFLAG or pCI-neo-^{3xFLAG}SARS-CoV-2-Nsp1 wildtype or mutants using Lipofectamine 3000 reagent (Cat. no. 18324012, Thermo Fisher

Scientific) in Opti-MEM Reduced Serum Medium (Thermo Fisher Scientific). After 16 h of transfection, newly synthesized proteins were detected using the Click-iT™ HPG Alexa Fluor™ 594 Protein Synthesis Assay Kit (Cat. no. C10429 Thermo Fisher). Briefly, cells were incubated in L-methionine-free DMEM (Cat. no. 21013, Thermo Fisher Scientific) for 30 min prior to the addition of 50 µM methionine analog L-homopropargylglycine (HPG) for 30 min. After fixation and permeabilization, the Click-iT reaction was performed using Alexa-488 according to the manufacture's protocol. Immunofluorescence was detected in a Zeiss Axiovert 200 M automated microscope using a 63X Plan-APOCHROMAT lens (1.4 numerical aperture) and captured by a Hamamatsu ORCA-spark CMOS monochrome camera. Image processing and analysis were performed as described in the ViewRNA methods section.

Generation of recombinant mutant SARS-CoV-2

We used our previously described BAC-based SARS-CoV-2 reverse genetic systems (4) to generate the rSARS-CoV-2 containing the D33K/E36K/E37K/E41K mutation in the nsp1. Primers (5'-gctcgtacgtggctttggaaaatccgtgaagaaagtcttatcaaaggcacgtcaacatc-3' and 5'-gacgtgcctttgataagactttcttcacggattttccaaagccacgtacgagcac-3') were used to introduce the D33K/E36K/E37K/E41K mutations into the pUC57-F1 shuttle plasmid by site-directed mutagenesis. Then, F1 was removed from the mutant pUC57 shuttle plasmid and used to substitute the F1 segment in the wild-type BAC. Lastly, the BAC containing the D33K/E36K/E37K/E41K mutations was prepared and moved to a biosafety level 3 (BSL3) laboratory at Texas Biomedical Research Institute to perform the transfection of Vero AT cells using lipofectamine 2000 according to the manufacturer's instruction as previously described (5). Transfected cells were monitored daily until complete cytopathic effect (CPE). The cell culture supernatant (P0) was collected, titrated, and used to infect monolayer of Vero AT cells at MOI of 0.001 to generate a P1 stock. The P1 stock was aliquoted, titrated, deep sequenced (Supplementary Fig. 2), and stored at -80 °C until being used.

SARS-CoV-2 growth kinetics

A549-ACE2 cells were seeded in 24-well format at a density of 100,000 cells per well. The next day, cells were infected at MOI 0.1 for 1h. Infections were performed in Viral Growth Media (VGM) (Dulbecco's modified Eagle's medium (Corning) supplemented with 2% FBS (Peak Serum), 1% non-essential amino acids (Gibco), 1% HEPES (Gibco) and 1% penicillin/streptomycin (Corning) at 37°C and 5% CO_2. Inoculum was removed and replaced with fresh infection media. Supernatants were collected at 12, 24, 36, and 48 hpi. Supernatants were stored at -80 ^{0}C before evaluation of viral titers. Titers were quantified by plaque assay in Vero E6 cells as previously described (6). Briefly, Vero E6 cells were seeded in 12-well format at a density of 365,000 cells/ well. The next day, confluent monolayers were infected with serial ten-fold dilutions of supernatants of infected cells for 1 hour at 37°C and 5% CO2 using an inoculum of 200 μL per well. Next, the inoculum was removed and an overlay consisting of MEM (Lonza) with penicillin/streptomycin (Corning), L-Glutamine (Gibco), HEPES (Gibco), BSA (MP Biomedicals), and $NaHCO_3$ supplemented with 0.7% purified agar (Oxoid) and 2% FBS (Peak Serum) was added to each well. Cells were stored at 37°C and 5% CO_2 for 72 h post-infection. Plaque assays were fixed with 4% formaldehyde overnight. Immunostaining was performed using anti-SARS-CoV-2 N monoclonal antibody (1C7C7, Creative Biolabs) for the primary antibody at a 1:1,000 dilution and anti-Mouse IgG (Abcam ab6823) for the secondary antibody at a 1:5,000 dilution. Plaques were developed using TrueBlue substrate (KPL Seracare).

SARS-CoV-2 infection *in vivo*

8-week-old female B6.Cg-Tg(K18-ACE2)2Prlmn/J mice were obtained from The Jackson Laboratory and housed in a BSL-3 vivarium at the Icahn School of Medicine at Mount Sinai under the guidelines of the Institutional Animal Care and Use Committee (IACUC) of the Icahn School of Medicine at Mount Sinai (ISMMS). Mice were intranasally mock-infected with sterile PBS or

infected with $1x10^4$PFU of either rSARS-CoV-2 $Nsp1^{WT}$ or rSARS-CoV-2 $Nsp1^{D33K/E36K/E37K/E41K}$ (n=10 and n=15 per group) in a 30 μl total inoculum. Infections were performed on animals anesthetized with ketamine (80 mg/kg) / Xylazine (12.5 mg/kg) given intraperitoneally prior to infection. Animals were monitored for morbidity and mortality for 10 dpi. At 4 and 7 dpi, mock-infected animals (n=7 at 4 dpi and n=4 at 7 dpi) or animal infected with rSARS-CoV-2^{WT} (n=7 at 4 dpi and n=4 at 7 dpi) or rSARS-CoV-$2^{D33K/E36K/E37K/E41K}$ (n=5 at 4 dpi and n=4 at 7 dpi) were euthanized with pentobarbital given intraperitoneally to perform necropsies. The left lung was taken for histology and stored in 10% formalin (Fisherbrand). After fixation for 72 h, tissues were transferred to PBS and sent for processing, paraffin embedding, sectioning, and H&E staining at the Biorepository and Pathology Core at ISMMS. All sections were assessed and evaluated by two board certified veterinary pathologists who reached consensus and were blinded to the treatment groups. Lung pathology was scored with ordinal semi-quantitative criteria as outlined by Table S3. Additional serial sections were utilized for immunohistochemistry (IHC) analysis using SARS-CoV-1/2 Nucleocapsid Protein (1C7C7) mouse monoclonal antibody diluted to 1:1,000 (Cell Signaling, Catalog number: 68344). A Ventana Discovery Ultra (Roche, Basel, Switzerland) tissue autostainer was used for chromogenic IHC. Because the primary antibody was of mouse origin, a mouse-on-mouse anti-Ig linking antibody (ab133469) was utilized followed by species-specific anti-rabbit secondary antibodies conjugated to horseradish peroxidase (Vector Laboratories, Burlingame, CA). Antigens were developed using the 3,3'-Diaminobenzidine (DAB) chromogen and counterstained with hematoxylin. Controls included the following: positive control tissue known to possess the antigen under investigation and negative controls derived from an uninfected murine lung specimen and an isotype antibody control known not to react with murine tissues (i.e., exclusive human specificity). Whole Slide Imaging and quantitative image analysis: chromogen-labeled slides were imaged using a Vectra Polaris Quantitative Pathology Imaging System (Akoya Biosciences) to generate whole slide images for each animal described in this study utilizing a brightfield acquisition protocol at 200x

magnification. View settings were adjusted to allow for optimal visibility of SARS-CoV-2 N protein and to reduce background signal by setting threshold gates to minimum signal intensities. After optimizing view settings, annotations around the entire tissue were created to define the analysis area using the flood tool and any tissue artifacts (i.e., folds, dust, etc.) were excluded using the exclusion pen tool. For quantification, the HALO (v3.6, Indica Labs, Albuquerque, NM, USA) Area Quantification (AQ) module was developed and fine-tuned to quantify viral antigen. Thresholds were set to define positive immunoreactivity based on a real-time tuning feature. AQ results were reported as the percentage of total area displaying positive immunoreactivity. HALO data was exported as an .csv file, with statistical analysis conducted using GraphPad Prism 10. The superior lobe, middle lobe, inferior lobe, and post-caval lobe of the right lung were taken for viral titers. Nasal turbinate was also taken for determining viral titers. The remaining animals were monitored for morbidity up to day 10 post-infection. For determination of viral titers tissue samples were thawed and homogenized 3 times at 4 M/S, centrifuged, and supernatants were processed as described above.

References

1. K. Zhang *et al.*, Nsp1 protein of SARS-CoV-2 disrupts the mRNA export machinery to inhibit host gene expression. *Sci Adv* **7** (2021).
2. J. C. Talian, J. B. Olmsted, R. D. Goldman, A rapid procedure for preparing fluorescein-labeled specific antibodies from whole antiserum: its use in analyzing cytoskeletal architecture. *The Journal of cell biology* **97**, 1277-1282 (1983).
3. A. Mor *et al.*, Influenza Virus mRNA Trafficking Through Host Nuclear Speckles. *Nat Microbiol* **1**, (7):16069. doi: 16010.11038/nmicrobiol.12016.16069. (2016).
4. C. Ye *et al.*, Rescue of SARS-CoV-2 from a Single Bacterial Artificial Chromosome. *MBio* **11** (2020).
5. C. Ye, L. Martinez-Sobrido, Use of a Bacterial Artificial Chromosome to Generate Recombinant SARS-CoV-2 Expressing Robust Levels of Reporter Genes. *Microbiol Spectr* **10**, e0273222 (2022).
6. F. Amanat *et al.*, An In Vitro Microneutralization Assay for SARS-CoV-2 Serology and Drug Screening. *Curr Protoc Microbiol* **58**, e108 (2020).
